# Identification of vaccine and drug targets in *Shigella dysenteriae* sd197 using reverse vaccinology approach

**DOI:** 10.1038/s41598-021-03988-0

**Published:** 2022-01-07

**Authors:** Khurshid Jalal, Tareq Abu-Izneid, Kanwal Khan, Muhammad Abbas, Ajmal Hayat, Sami Bawazeer, Reaz Uddin

**Affiliations:** 1 H.E.J. Research Institute of Chemistry International Center for Chemical and Biological Sciences, University of Karachi, Karachi, Pakistan; 2 Pharmaceutical Sciences, College of Pharmacy, Al Ain University Al Ain Campus, Al Ain, United Arab Emirates; 3 Lab 103 PCMD Ext. Dr. Panjwani Center for Molecular Medicine and Drug Research, International Center for Chemical and Biological Sciences, University of Karachi, Karachi, 75270 Pakistan; 4 Department of Pharmacy, Abdul Wali Khan University Mardan KP, Mardan, Pakistan; 5 Pharmacognosy Department, College of Pharmacy, Umm Al-Qura University, Makkah, Saudi Arabia

**Keywords:** Computational biology and bioinformatics, Drug discovery

## Abstract

Shigellosis is characterized as diarrheal disease that causes a high mortality rate especially in children, elderly and immunocompromised patients. More recently, the World Health Organization advised safe vaccine designing against shigellosis due to the emergence of *Shigella dysenteriae* resistant strains. Therefore, the aim of this study is to identify novel drug targets as well as the design of the potential vaccine candidates and chimeric vaccine models against *Shigella dysenteriae*. A computational based Reverse Vaccinology along with subtractive genomics analysis is one of the robust approaches used for the prioritization of drug targets and vaccine candidates through direct screening of genome sequence assemblies. Herein, a successfully designed peptide-based novel highly antigenic chimeric vaccine candidate against *Shigella dysenteriae* sd197 strain is proposed. The study resulted in six epitopes from outer membrane WP_000188255.1 (Fe (3+) dicitrate transport protein FecA) that ultimately leads to the construction of twelve vaccine models. Moreover, V9 construct was found to be highly immunogenic, non-toxic, non-allergenic, highly antigenic, and most stable in terms of molecular docking and simulation studies against six HLAs and TLRS/MD complex. So far, this protein and multiepitope have never been characterized as vaccine targets against *Shigella dysenteriae*. The current study proposed that V9 could be a significant vaccine candidate against shigellosis and to ascertain that further experiments may be applied by the scientific community focused on shigellosis.

## Introduction

*Shigella* spp. is a facultative anaerobic, non-motile, and gram-negative bacterium that causes severe diarrhea and dysenteric disease called shigellosis or bacillary dysentery^1^. *Shigella* spp. infects the gastro-intestinal tract of humans and primates resulting in symptoms of fever, abdominal cramps, and watery, or bloody diarrhea. Recently, a study reported that the third prominent reason for worldwide mortality in infants is diarrhea^2^. There are four serogroups of *Shigella*: *S. dysenteriae* (15 serotypes)*, Shigella flexneri* (~ 18 serotypes), *S. sonnei* (one serotype), and *S. boydii* (20 serotypes). These all serogroups are associated with shigellosis in humans^3^. The Centers for Disease Control (CDC) and Prevention reported that an estimated 80–165 million cases and 0.6 million deaths worldwide are caused by *Shigella spp*. annually, once causes pandemic dysentery in South Asia and Sub-Saharan Africa^4^.

The infection mechanism of *Shigella* spp begins with the binding and entrance into the epithelial cell of the intestine with the help of exposed needle like structure known as type three secretory system (T3SS) formed by Spa and Mxi proteins. The proteins secreted by T3SS, cooperatively with some other effector proteins such as invasion plasmid antigens (IpaA, IpaB, IpaC, and IpaD) and IcsA/VirG facilitate invasion and intercellular spread of *Shigella* to the adjacent cells^5^. Among the Ipa effector proteins, IpaB controls the T3SS and helps in escape from macrophages contributing as an important virulent factor. *Shigella dysenteriae* also used another type of invasion mechanism that works on release on toxins such as AB_5_ toxin, composed of one A and five B subunits. The A subunit of Shiga toxin is responsible for counteracting the enzymatic activity as it permanently inactivates the ribosome of the host cell, and wind up all protein synthesis processes. The penetrating process begins when the B subunits of Shiga toxin bind to the host cell surface receptor, globotriaosylceramide (Gb3) initiates an uptake mechanism by the host cell, and eventually, the toxin will completely be entered to the cytoplasm of the host cell. When the toxin reaches its final destination, the A subunit can separate from the B_5_ subunit and brings out its function^6^.

The emergence of antibiotics resistant strains of *Shigella dysenteriae* has caused alarming situation regarding shigellosis treatment around the globe^7^. Early evidence revealed that the attenuated bacterial vaccines may have the ability to stimulate immune system and previously used against *Shigella dysenteriae*^8^. However, all those vaccines have shown limitations due to instability, limited protection period, and reactogenicity^9^. On the other hand, the new drug discovery is of prime importance for the treatment of shigellosis. Identification of novel drug targets is one of the best approaches in drug discovery pipeline. Nevertheless, the screening of thousands of macro-molecules and their subsequent in vivo assays in wet lab are highly time and money consuming approaches. However, developments in computational biology and various other bioinformatics fields have made wonderful progress that are leading to reduction in time and money for the purpose of drug discovery^10^. Computational based method typically utilizes alternate approaches for finding novel drug targets and designing multi-epitopes vaccines, designing structure-based drugs, elucidating the host–pathogen interactions, allowing genome-based comparative study, and so on thereby reducing the conventional laboratory-based experimental practices^11^.

Reverse Vaccinology (RV) is one of the powerful and novel in silico vaccine designing approaches originally developed to overcome the limitations of current vaccinology methods. The RV has been widely applied against numerous deadly pathogens resulting in the development of first successful *Neisseria meningitidis* serogroup B, MenB vaccine^12^. Simultaneously, conventional laboratory-based approaches are frequently failed to deliver an efficient and universal vaccine especially for affected groups of patients. Although various vaccine candidates are in different trials against shigellosis, yet no prophylactic or therapeutic vaccine is available in the market^8,13^. Therefore, there is an urgent need to identify novel drug targets and find a new and reliable vaccine model to fight against shigellosis.

Herein, the aim was to predict potent drug targets using subtractive genomics and potential immune cells epitopes in *Shigella dysenteries* Sd1967 strain that can trigger B and T-cell immune responses, using immunoinformatic Reverse Vaccinology approach. A Reverse Vaccinology approach must be used against this deadly pathogen to model chimeric vaccine model that can induce broad-spectrum humoral as well as cellular immunity. The RV protocol is comprised of numerous in silico filters that can prioritize high probability proteins as vaccine candidates from whole proteome of pathogen. We strongly believe that the outcome of the current study may further facilitate the production of vaccine constructs through experimental (in vivo and in vitro) studies against *Shigella dysenteriae*.

## Materials and methods

**Figure 1:**
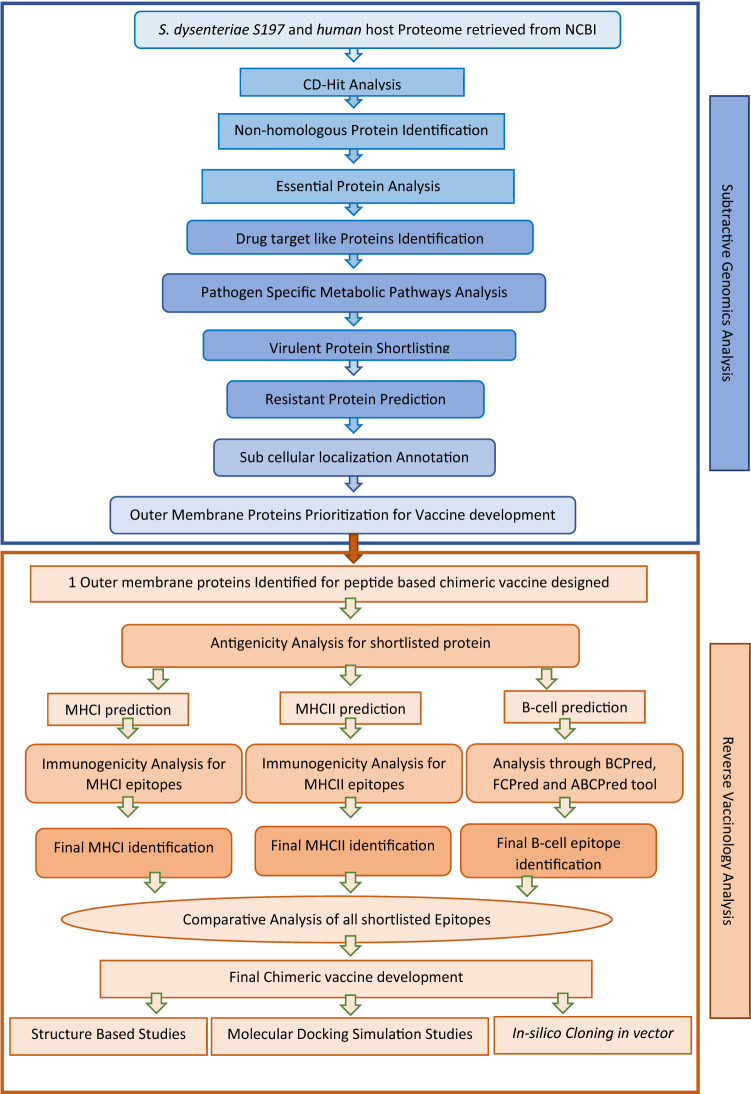
Flow chart for Current study. (A) Subtractive Genomics and Reverse Vaccinology. Flowchart of proposed study for peptide based chimeric vaccine constructs.

The current study applied the subtractive genomic approach to prioritize potential drug targets along with Reverse Vaccinology approach that is a computational based method for the identification of vaccine candidates against *Shigella dysenteriae*. The flow chart (Fig. 1) has summarized the protocol used for the identification of multi-epitopes based chimeric vaccine candidates.

### Data retrieval

The Kyoto Encyclopedia of Genes and Genomes (KEGG) database^14^ was used for the retrieval of metabolic pathways of pathogen (i.e. *Shigella dysenteriae Sd179)* and host (i.e. *Homo sapiens hsa)*, while whole proteome of *Shigella dysenteriae* Sd179 (Serotype 1) was obtained from National Center for Biotechnology Information (RefSeq NCBI)^15^ i.e. GCF_000012005.1. On the other hand, the Universal Protein Resource (UniProt) database^16^ was used for the retrieval of human proteome (UniProt ID: UP000005640). The essentiality of drug target was assessed by the Database of Essential Genes (DEG database) while the DrugBank database was used to find the druggability potential of the shortlisted protein based drug targets.

### Finding non-paralogous sequences

The non-paralogues proteins were identified by using Cluster Database at High Identity with Tolerance (CD-HIT) database^17^. The proteins that have more than 80% similarities with other proteins are paralogous (the set threshold was 0.8), and subsequently were eliminated, resulting only in the identification of non-paralogous proteins.

### Non-homologous protein identification

Consequently, the resulted protein sequences were retrieved from the CD-HIT analysis and subjected to BLASTp with a cut-off of E-value 10^−3^ against the whole proteome of *Homo sapiens*. The BLASTp resulted in ‘Hits’ (Homologous sequences) proteins having > 80% similarities with humans and ‘No Hits’ (Non-homologous sequences) having no similarities at all. For further analysis, non-homologous (No hits) sequences were selected and retrieved to avoid the functional and structural similarities with the human proteins in order to minimize the cross-reactivity.

### Prediction of non-homologous essential proteins

The proteins having a key role in cellular metabolisms are meant to be essential for an organism’s survival. Thus, a BLASTp of non-homologous *Shigella dysenteriae* proteins was performed to shortlist proteins essential to the pathogen’s survival against Database of Essential Gene (DEG)^18^ with *E-*value 10^−5^. The DEG is comprised of experimentally identified essential gene products. The protein sequences of *Shigella dysenteriae* have significant sequence similarity with the DEG proteins. Therefore, the significant similar sequences were retrieved for further analysis, and the remaining non-similar proteins were excluded.

### Druggability of essential proteins

Similarly, essential nonhomologous proteins were evaluated through BLASTp with E-value 10^−5^ against DrugBank database^19^ to determine their drug target like ability to finally identify novel drug targets. The essential proteins were analyzed against the customized FDA approved dataset of drug targets. Proteins having high similarity frequency (80% or more) with a database for FDA approved DrugBank database were considered as druggable target.

### Host and pathogen metabolic pathway investigation

The essential, drug target like and non-homologous pathogen proteins obtained from the previous work was subjected to KEGG^20^ database using KAAS server^21^. A standalone comparison was performed between the host and pathogen to identify unique and commonly found metabolic pathways and their proteins. Unique pathways are classified as those pathways that are present only in the pathogen, whereas common pathways are considered as those that are present in *Shigella dysenteriae* (pathogen) as well as in human host. The protein sequences belonged to the unique metabolic pathways of *Shigella dysenteriae* were retrieved from the NCBI database for further analysis.

### Determination of virulence factor (proteins)

The pathogen synthesizes certain chemical molecules that help bacteria to modulate infection in the host body and these are classified as virulent factors for pathogens. The virulence factors help in the adhesion, colonization, and invasion of the bacteria within the host to progress the disease. Consequently, a VFDB (Virulence Factor of Pathogenic Bacteria) database was used for the identification of virulence proteins^22^. The shortlisted proteins from the previous step were subjected to the BLAST against ‘VFDB Core set A’ proteins with a cut-off value of 0.0001 to find the virulence of the proteins for further investigation.

### Resistance proteins analysis

Various challenges are associated with the treatment of infectious diseases because of the significant rise in drug resistance and decrease in the potency of the antibiotics. The ARG-ANNOT V6 (Antibiotic Resistance Gene-ANNOTation V6) tool^23^ was used for the identification of novel resistance protein sequences from the whole proteome of the pathogen. It consists of experimentally proved protein sequences playing roles in resistance to various classes of antibiotics such as fluoroquinolones, beta-lactamases, aminoglycosides, trimethoprim, glycopeptides, fosfomycin, rifampicin, and sulfonamide^23^. The shortlisted protein FASTA sequences were then BLAST against the resistance proteins of the ARG-ANNOT V6 database with a threshold of 10^–524^.

### Sub-cellular location prediction

Determination of the subcellular localization of proteins is important for accurate and reliable drug target identification and multi-epitopes vaccine construction. In the current study, the subcellular localization of proteins was investigated through bioinformatics tool such as PSORTb version 3.0.2^25^. This tool determines the subcellular localization of proteins based on the amino acid sequence. The subcellular localization results may consist of cytoplasm, cytoplasmic membrane, cell wall, extracellular and unknown protein localization.

### Prioritization of antigenic proteins

Antigens are the molecules that recognized by the host immune system to induce immune response. Though intracellular proteins may act as drug targets, yet it has been widely studied that membrane proteins that confer immunity are more preferable for vaccine candidates. The VaxiJen v2.0 server was used with default parameters of 0.5 to determine the most potent antigenic proteins from whole *Shigella dysenteriae* proteome^26^.

### Prediction of MHC-I T-cell epitope

In order to generate immune memory cells against *Shigella dysenteriae*, the potential peptides of that activate human immune system should be identified. Various types of epitopes were determined from shortlisted surface proteins to understand the immunomodulatory effects through NetCTL server^27^ with a default parameter of 0.75. Furthermore, the combined scores of TAP transporter associated efficiency, proteasomal cleavage, and MHC-I affinity predictor were attained as a collective score for all the predicted epitopes^28^.

Additionally, Immune Epitope Database Analysis Resource (IEDB AR) was used to predict the binding of shortlisted epitopes with MHC-I^29^. The T-cell recognized epitopes were represented by MHC Class-I molecules. The default parameters of ANN, SMM, CombLib, and NetMHCpan along with human MHC and all HLA alleles were selected for the epitope prediction in the current study^29^. The T-cell epitopes with HLA alleles were shortlisted based on IC_50_ values and percentile ranks.

### Immunogenicity prediction of MHC-I epitopes

The MHC class 1 antigenicity was predicted through online bioinformatics server IEDB^30^. The T-cell epitopes must have certain immunogenic features that are capable of triggering the stimulation of either CD4 or CD8 T-cells. The epitopes with positive values were selected for further analysis.

### Antigenicity, conservancy, and toxicity analysis

Conservancy, toxicity, and antigenic properties were also analyzed for shortlisting MHC I immunogenic epitopes. Conserved sequence among all the genotype of MHC Class 1 epitopes was predicted via online tool IEDB conservancy analysis^31^ by implementing default parameters. The valuation of toxicity level was predicted by online tool ToxinPred by using a default parameter^32^. The antigenic nature of the shortlisted peptides was further assessed through VaxiJen online server having an accuracy of 70–80%, and 0.5 probability threshold scores for antigenic properties were used^31^.

### T-cell MHC II prediction

The MHC class 2 epitopes were determined through IEDB-AR server based on consensus methods using average relative binding matrix method (BMM) and stabilization matrix alignment method (SMM)^33^. The top binders with the cut-off value of < 0.2 peptide rank and IC_50_ < 100 nM were selected against the 95% HLA variability found in the global human population i.e., HLA-DRB4 ∗ 01:01, and DRB1*1501, DRB1*0401, DRB1*0701, DRB3 ∗ 01:01, DRB1*0801, DRB1*1301, DRB1*0101, DRB1*0301, DRB1*1101, HLA- HLA-DRB3 ∗ 02:02, HLA-DRB5 ∗ 01:01,^34^.

### MHC I and II restricted alleles cluster analysis

Importantly, MHCcluster v2.0 was used to validate the predicted T-cell epitopes through clustering the predicted MHC I-II restricted allele from IEBD analysis resource with their appropriate peptides. It results in graphical trees and a static heat-maps generation that explains the functional correlation between the peptides and HLAs.

### B-cell epitopes prediction and construction of model vaccine

The online server BCPREDS, i.e., B-cell Epitope Prediction Server (http://ailab.ist.psu.edu/bcpred/predict.html)^35^ FBCpred^36^ and ABCpred^37^ were used for the prediction of B Cell epitopes. The BCpred works on five different kernel methods whereas FBCpred is based on consequent kernel methods for the prediction of epitopes. The cut-off values used for the identification of B cell epitopes with these were set as > 0.8^38^ while the predictions were classified based on biochemical properties, hydrophilicity, hydrophobicity, surface accessibility, amino acids sequences, and secondary structure. Furthermore, ElliPro server^39^ of IEDB was also used to further characterize B-cell epitopes on the basis of hydrophobicity, flexibility with Emini surface accessibility prediction tool^40^, antigenicity, accessibility^41^, Chou and Fashman beta-turn prediction tool^42^, respectively. Finally, the identified B-cell epitopes were mapped against T-cell epitopes manually. It resulted in the common epitopes found in B cell and T cell as the most probable B-cell epitopes that were used further for vaccine designing.

Consequently, a different combination of shortlisted B-cell epitopes was analyzed to construct the vaccine model having low toxicity, allergenicity, and high immunogenicity. The resulted vaccine construct was used for further analysis.

### Antigenicity, solubility, and allergenicity assessment for vaccines constructs

Nowadays, vaccines are reported for the production of adverse allergic reactions. In order to overcome the allergic features of vaccine model, AlgPred tool was used to examine the allergenicity of model vaccine sequences with a cut-off score of −0.4 and 85% accuracy^43^. Scores less than the cut-off value were considered non-allergenic vaccines. The VaxiJen and ANTIGEN servers were used for the prediction of antigenicity of vaccine construct by using the threshold value of > 0.5^44^. The constructed vaccine should be soluble upon the expression in *E. coli*. To check the solubility, SOLpro program was used to predict vaccine models solubility with an overall accuracy of 74% at corresponding probability (≥ 0.5).

### Physicochemical properties analysis

The vaccine constructs were functionally characterized by investigating the physicochemical properties by using Expasy ProtParam online server (http://expasy.org/cgi-bin/protpraram)^45^. The physicochemical properties are consisting of molecular weight, pK values of different amino acids instability index, GRAVY values, isoelectric pH, hydropathicity, approximate half-life of generated vaccine model, and aliphatic index of the constructs^46^.

### Comparative structure modelling

The 3-dimensional (3D) structure of vaccine construct was achieved by^47^ SWISS-MODEL structure modeling server^48^. The PSIPRED^49^ was used for the secondary structure evaluation while PROCHECK for tertiary evaluation^50^.

### Molecular docking studies

Molecular docking of final vaccine model with six different Human Leukocyte Antigen (HLA) alleles was performed through a bioinformatics tool PatchDock^51^ to find the interactions. These six HLA alleles were retrieved from PDB database with PDB IDs: 3C5J (HLA-DR B3*02:02), 2FSE (HLA-DRB1*01:01), 2Q6W (HLA-DR B3*01:01), 1H15(HLA-DR B5*01:01), 1A6A (HLA-DR B1*03:01) and 2SEB (HLA-DRB1*04:01). The FireDock (Fast Interaction Refinement in Molecular Docking) server was implemented for further refinement and validation of interactions obtained from the PatchDock. Likewise, the docking of vaccine with TLR4 (PDB ID: 2Z65) was performed using PatchDock server and refined through FireDock tool. Furthermore, the docking step was validated by GRAMMX tool^52^ for vaccine and TLR4/MD complex based on accuracy score, interactions similarity score, and hydrogen bonding pattern. The UCSF Chimera^53^ and PDBsum^54^ were used for the interpretation of the best model of vaccine complex visualization and interactions.

### Molecular dynamics simulation

The stability and flexibility of vaccine constructs in physiological environment were determined through GROMACS server according to published method^55^. The solvation was accomplished with Simple Point Charge (SPC) water model, with steepest energy minimization algorithm while NVT and NPT were ensembled for 50,000 steps (100 ps) at 1 atm pressure and 300 K. Eventually, the vaccine Molecular Dynamics Simulation was accomplished for 10 ns. Likewise, Molecular Dynamics Simulation of docked complex (vaccine with TLR4) was also performed via iMODs server, a fast and free-accessible server being used for the identification of complex stability and flexibility in terms of covariance, B-factors, eigenvalue, and deformability.

### Immuno-simulation of constructed vaccine

The vaccine immune-simulation and response of the immune system was examined through bioinformatics tools C-ImmSim simulation server^56^. The vaccine was administered at three different intervals for four weeks while keeping all the simulations at default with periods set at 1, 82, and 126 (according to 8 h correspond to one cell division cycle in real life^56^) and random seed at 12,345 with vaccine injection that does not consist of LPS (lipopolysaccharide). The volume and the steps of the immuno-simulation were set at 10, and 1000, respectively, with homozygous host haplotypes HLA-B*0702, HLA-DRB1*0101, HLA-A*0101, HLA-A*0201, and HLA-DRB1*0401.

### In silico cloning and codon optimization of final vaccine construct

The JCAT (Java Codon Adaptation Tool) was used to back translate the vaccine sequences into cDNA in order to improve the expression of constructed vaccine proteins in *E. coli* system^57^. The JCAT tool was used to determine the GC contents of DNA sequence and Codon Adaption Index score (CAI) for the optimized nucleotide sequence while avoiding the prokaryotic ribosome binding sites and Rho-independent termination of transcription cleavage site for restriction enzymes. The pET-28a( +) vector was used to ensure the vaccine construct cloning and expression in *E. coli* using Snapgene tool.

## Results

### Subtractive genomics approach for drug targets identification

The subtractive genomics is a powerful computational method used to gradually reduce the whole proteome and metabolic pathways of the pathogen providing necessary information for a set of proteins crucial to micro-organisms but absent in the respective host. Subtractive genomics plays a pivotal role in novel drug target identification which is unique and essential for the survival of the pathogen without altering the host (human) biochemical pathways. It employs the identification of non-paralogues, non-homologous to human proteome, essential, druggable, virulent, and resistant proteins as discussed below.

### Determination of non-paralogous proteins

In order to prioritize the non-paralogous proteins, the CD-HIT analysis was performed. Out of 3361 proteins (whole proteome of *Shigella dysenteriae* was shown in Table 1), the 3173 proteins were non-paralogous which were analyzed further and the remaining 188 paralogous proteins were subsequently discarded.

**Table 1:** Complete proteome of *Shigella dysenteriae* sd197.

Strain ID	Strain names	Proteins	Pathways
GCF_000012005.1	Sd197	3361	108

### Prioritization of non-homologous proteins

The BLAST was run with the non-paralogous proteins against the whole proteome of humans to determine the non-homologous nature of the vaccine using the cut-off value of 0.0001 (E-value 10^–3^). The BLASTp results revealed that only 2275 proteins were non-homologous to the human while rest of the 898 proteins were homologous to the human and consequently discarded.

### Identification of essential proteins

The resulted 2275 non-homologous proteins were further subjected to BLAST against the DEG (Database of Essential Gene) to determine the essentiality of these proteins. The threshold was set as E-value 10^−5^. The DEG results showed that 1532 proteins were essential for the survival of *Shigella dysenteriae*. These essential proteins can be used for the designing of novel drug targets.

### Druggability analysis of shortlisted proteins

The shortlisted essential, non-homologous proteins were then subjected to BLAST against the DrugBank database to find the druggable characteristics. The DrugBank database results revealed that out of 1532 proteins only 413 proteins showed druggability potential. Therefore, only 413 proteins were proceeded to further analysis.

### Pathogen-specific metabolic pathways analysis

The KEGG Automatic Annotation Server (KAAS) was used for the analysis of human-host and *Shigella dysenteriae* metabolic pathways. Both the human and *Shigella dysenteriae* metabolic pathways with their recommended IDs were retrieved from the KEGG. *Shigella* is comprised of 108 metabolic pathways that are responsible for all metabolic processes. In comparison, humans are comprised of 336 metabolic pathways. A standalone manual comparison was performed to compare both the host and pathogen pathways. It resulted in 82 common pathways (i.e. common to both host and pathogen) while 25 pathways (Table 2) were unique only to *Shigella dysenteriae*. The unique pathways are consisted of 422 proteins which are required to further compare with the druggable proteins. The results showed that only 128 proteins were found similar in both categories i.e. (1) the druggable proteins and (2) unique metabolic pathway proteins.

**Table 2:** Unique metabolic pathways found in *Shigella dysenteriae.*

S. no	Pathways	Pathway IDs	No of proteins
1	Benzoate degradation	Sdy00362	4
2	Aminobenzoate degradation	Sdy00627	4
3	Fluorobenzoate degradation	Sdy00364	1
4	Chloroalkane and chloroalkene degradation	sdy00625	2
5	Chlorocyclohexane and chlorobenzene degradation	sdy00361	1
6	Toluene degradation	sdy00623	1
7	Nitrotoluene degradation	sdy00633	5
8	Two-component system	sdy02020	112
9	Quorum sensing	sdy02024	54
10	Bacterial chemotaxis	sdy02030	8
11	Flagellar assembly	sdy02040	22
12	Beta-Lactam resistance	sdy01501	18
13	Vancomycin resistance	sdy01502	6
14	Cationic antimicrobial peptide	sdy01503	31
15	Ribosome	sdy03010	75
16	Carbapenem biosynthesis	sdy00332	2
17	Monobactam biosynthesis	sdy00261	8
18	Streptomycin biosynthesis	sdy00521	8
19	Acarbose and validamycin biosynthesis	sdy00525	3
20	Novobiocin biosynthesis	sdy00401	4
21	Caprolactam degradation	sdy00930	2
22	Ethylbenzene degradation		2
22	Naphthalene degradation	sdy00626	2
24	Limonene and pinene degradation	sdy00903	2
25	Geraniol degradation	sdy00281	5

### Virulence proteins examination

The virulence proteins are responsible for the bacterial survival in the host cell by overcoming the host immune system. The VFDB database provides complete information on protein virulence. The VFDB results showed that out of 128 proteins 99 proteins were associated with the virulence of *Shigella dysenteriae*. Conversely, these 99 proteins were further studied to identify potent vaccine targets.

### Resistance proteins identification

The resistance proteins are those which are responsible for the expulsion of the antibiotics from the bacterial cell to bypass the drug action. The virulent proteins of *Shigella dysenteriae* were determined through BLAST against the ARG-V6 database. The results revealed that out of 99 proteins, 96 proteins were associated with the resistance of *Shigella dysenteriae*. These resistance proteins can be used as a potent drug as well as a vaccine target.

### Proteins subcellular localization prediction and target prioritization

**Figure 2:**
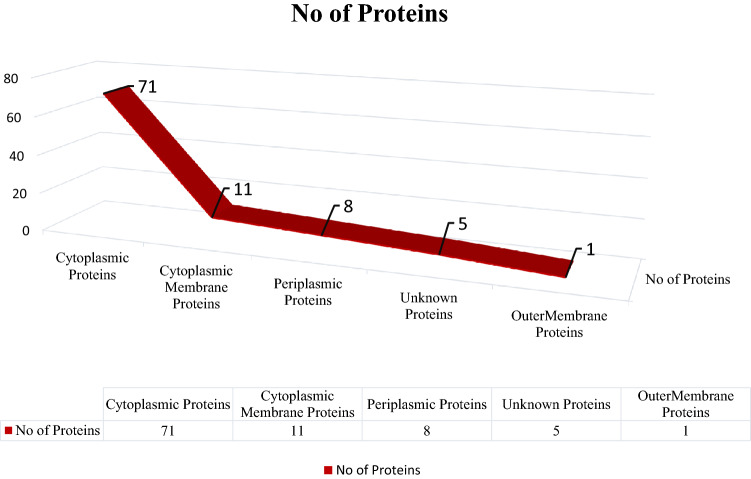
Sub-cellular Localization. Quantitative representation of sub-cellular localization of shortlisted essential, druggable, pathogen specific proteins predicted through PsortB.

The sub-cellular localization identification is one of the crucial steps to reduce time, labor, and resources for the identification of best vaccine target and therapeutic agents designing. Hence, the shortlisted 96 non-paralogous, non-homologous essential proteins were screened through PSORTb tool for the prediction of subcellular localization. Among these, 71 proteins were associated with the cytoplasmic proteins, 11 proteins found in the cytoplasmic membrane, 8 proteins were associated with periplasm. Nevertheless, there are few protein sequences (i.e. 5) found as unknowns and 1 protein i.e. WP_000188255.1; Fe (3+) dicitrate transport protein (FecA) was identified as outer-membrane (highlighted in Fig. 2). The identified outer-membrane FecA protein was used to construct a multi-epitope vaccine. Significantly, the identified localization of all these 96 druggable targets filtered through the subtractive genomics criteria helped to minimize the time, and resources to optimize the best drug/vaccine targets against *S. dysenteriae*. The Supplementary Table S1 highlighted the identified drug targets and vaccine candidates that can be used to target *S. dysenteriae* and helps to target shigellosis.

### Significance of selected protein

FecA is the outer membrane receptor protein in the Fe(3+) dicitrate transport system. FecA protein is a part of TonB dependent transportation found in outer membrane (OM) of a pathogen (mostly gram-negative). It is studied that FecA is responsible for the transportation of diferric dicitrate (DFDC) siderophore across the OM. It plays main role to transmit a signal across the periplasmic membrane and activates other Fec proteins i.e. FecI, and FecR. This complete cascade of signaling i.e. signal, the signal receptor, transfer of the signal across the outer membrane, and the cytoplasmic membrane involves Fec transport gene regulation system. These FecA depends on TonB transportations mechanism provides a high affinity for iron-chelating siderophores, vitamins B_12,_ and other iron containing nutrients from the environment. It also transmits the regulatory signal across the outer membrane, contacting TonB through its TonB box, a heptapeptide at the carboxy-terminal border of its amino-terminal external extension. Hence, the diverse role of FecA in transportation mediates it as a suitable drug target as well as a vaccine candidate against *Shigella dysenteriae*^58^.

### Reverse vaccinology approach

The conventional vaccinology procedures have been transformed into Reverse Vaccinology due to the recent advancement of vaccinomics and allied omics methods^59^. It is one of the emerging computational approaches that has been used extensively to optimize the prediction of drug and vaccine targets usually for those pathogens that are difficult to grow in the laboratory. The Reverse Vaccinology strategy, which is part of the vaccinomics regime, uses bioinformatics approaches to scrutinize the complete genome of pathogens for genes that could lead to excellent epitopes, peptides in an antigen to which antibodies bind, and surface-located proteins. After that, the chosen proteins/peptides are synthesized and evaluated in animal models.

### Prioritization of antigenic proteins

The host-immune system induces a response by exposure to molecules termed as antigens. It has been widely studied and reported that outer-membrane proteins that confer immunity are more suitable for vaccine candidates and conversely, intracellular proteins are considered more suitable as novel drug targets. The antigenicity analysis for predicted outer-membrane proteins through VaxiJen v2.0 server was estimated to be 0.65 using a cut-off value of 0.5^26^.

### MHC class I T-cell epitope prediction

In order to attain the T-cell epitopes, the sequences of shortlisted outer-membrane protein were subjected to NetCTL server^60^. The NetCTL results showed that total of 766 T-cell epitopes peptides was generated. The IEBD server generated 21,000 MHC1 epitopes from these predicted T cell epitopes. From there only 199 epitopes were shortlisted by using the percentile rank of >  = 0.2. All of these shortlisted epitope peptides were recognized as having optimal binding affinity to T-cells and were evaluated further.

### MHC class 1 epitopes immunogenicity prediction

The epitopes present on the MHC Class-I molecules were identified by CD + 8 to detect distortion such as an infection. Several studies reported that immunogenicity of the peptide is dependent upon the amino acid sequence. A higher number of aromatic amino acids present in the peptides are more immunogenic than other peptides. The robustness of the interaction between the peptide-MHC complexes (pMHC) and T-cell receptor (***TCR***) depends on the presented peptide. The proficiency of epitopes to induce T-cell responses is based on the level of immunogenicity score. The shortlisted T-cell epitopes were examined for immunogenicity prediction using the cut-off value of the positive score. We select only those epitopes which had appositive values while excluded the negative values. The IEDB results showed that out of 199 epitopes 77 epitopes were classified as most immunogenic.

### Antigenicity, conservancy, and toxicity analysis

The online tool called ToxinPred was used for the evaluation of toxicity level. It revealed that all the shortlisted 77 epitopes were not toxic (do not cause any harm) to the host cell. Consequently, these epitopes were selected in next steps of Reverse Vaccinology. Similarly, IEDB conserved sequence analysis tool result showed that all the subjected epitopes were 100% conserved within the *Shigella dysenteriae* sd197. These conserved and non-toxic epitopes were then subjected to VaxiJen tool for the analysis of antigenicity with a cut-off value of 0.5. The VaxiJen result showed that out of 77 epitopes the 15 epitopes were more antigenic and selected for further evaluation while the rest of 45 less immunogenic epitopes were discarded as shown in Supplementary Table 2.

### Prediction of MHC-II epitopes

Additionally, the shortlisted outer membrane protein was also used to identify MHC Class-II epitopes using IEDB server. The epitopes having binding affinity < 200 nM and percentile ranks < 0.2 were shortlisted and used for further examination. The results showed that a total of 11,456 epitopes were generated. However, only five epitopes were shortlisted by applying the cut-off value of > 0.2.

### MHC restriction cluster analysis

**Figure 3:**
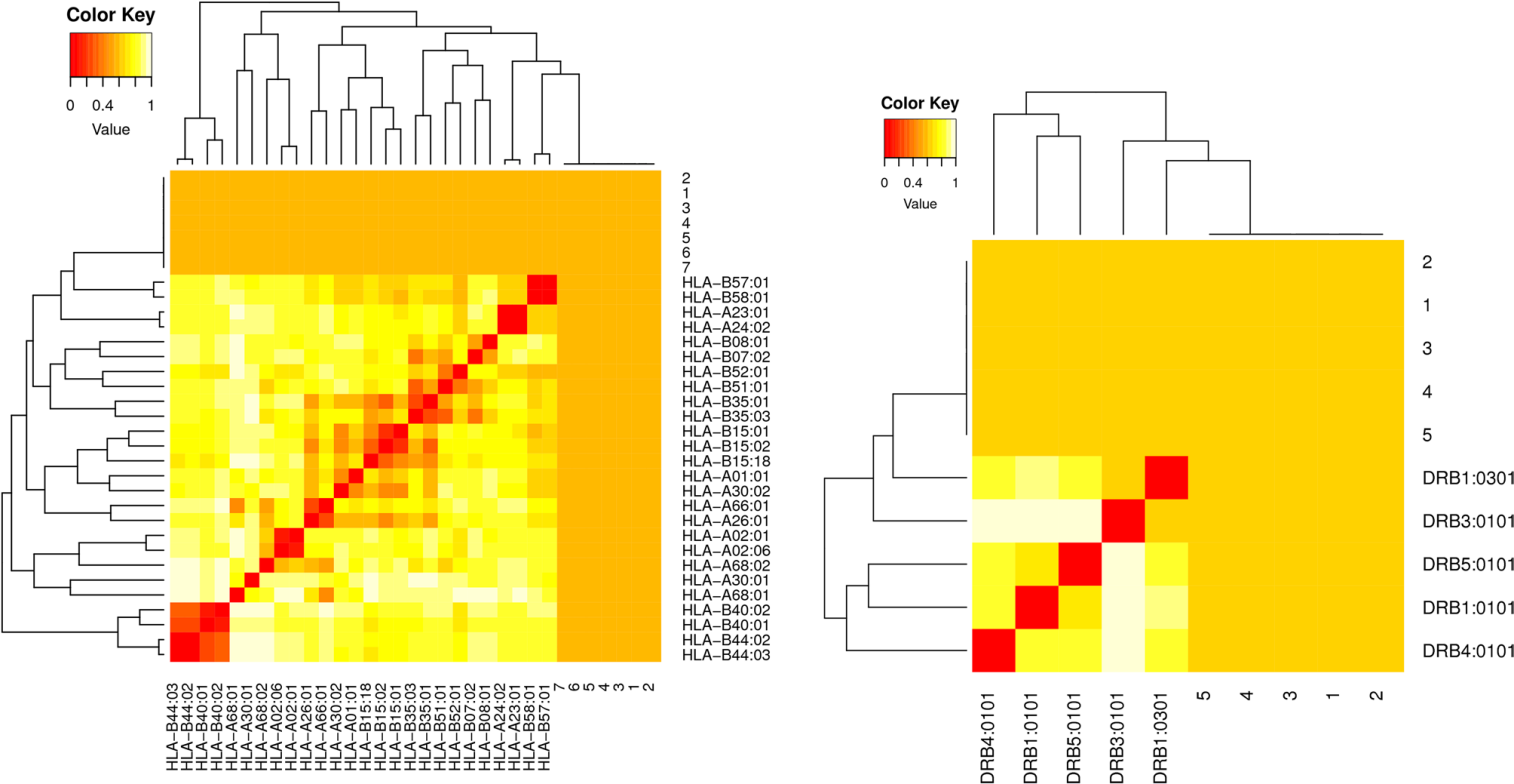
Clustering Analysis for MHC I and II epitopes. The Cluster analysis of MHC molecules and HLA alleles (**A**), MHCI clustering alleles, (**B**) MHCII clustering alleles. Red color indicating strong interaction while yellow zone indicates the weaker interaction.

The clusters of MHC restricted allele and their appropriate peptides were re-evaluated by cluster analysis. It resulted in the construction of heat map and phylogenetic tree (Supplementary Fig. 1) of MHC-1 and MHC II, respectively. Epitopes clustered are formed on the basis of their interactions with the Human Leukocyte Antigen (HLA). The yellow color represents weaker interaction while the red color shows strong interaction with proper annotation (Fig. 3).

### B-cell epitope prediction

**Figure 4:**
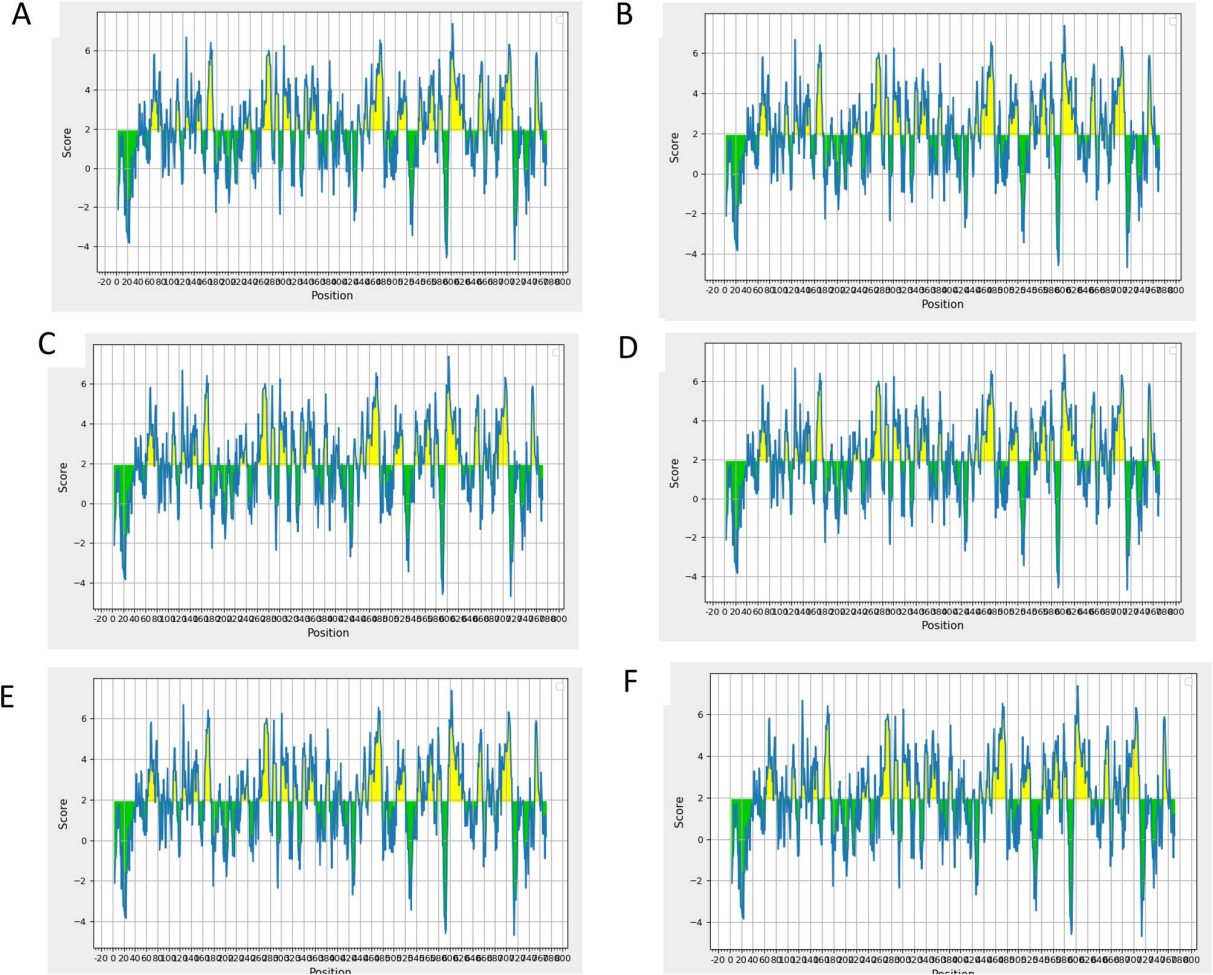
B-cell epitopes Analysis. (**A**) Bepipred Linear Epitope, (**B**)Chou &amp; Fasman Beta-Turn Prediction, (**C**) Emini Surface Accessibility Prediction, (**D**) Karplus&amp; Schulz Flexibility Prediction, (**E**) Kolaskar &amp; Tongaonkar Antigenicity, (**F**) Parker Hydrophilicity Prediction.

The MHC-I and MHC-II epitopes (cellular immunity), B-cell epitopes were also predicted using different online tools to induce humoral immunity. In order to eliminate the pathogen, humoral immunity is also necessary besides cellular immunity. The prediction and classification of B-cell epitopes play a vital role in vaccine designing, immunodiagnostic tests, and antibody production. The results showed that 20 epitopes were generated via BCpred server, 86 epitopes were generated through the FBCpred tool and 27 epitopes were predicted via ABCpred server. Moreover, resultant B-cells epitopes were further examined and shortlisted on the basis of BepiPred linear epitope prediction (Fig. 4A), Chou-Fasman beta-turn prediction (Fig. 4B), Kolaskar Tongaonkar antigenicity (Fig. 4C), Emini surface accessibility prediction (Fig. 4D), Karplus-Schulz flexibility prediction (Fig. 4E), and Parker hydrophilicity prediction (Fig. 4F). Furthermore, we compared all the epitopes generated by BCpred, FBCpred, and ABCpred in order to finalize the similar epitopes predicted through these tools. The result revealed that 26 epitopes were similar among these three tools (Table 3), and were used for further analysis.

**Table 3:** Comparative analysis of B Cell, MHC-I and MHC-II epitopes.

S. No	B-cell epitopes	MHC-I	MHC-II
1	LRVFR	WVRGIEPRY	LRVFRKTTPLVNAIR
2	AAQVNIAPG	MRFEHIESY	RVFRKTTPLVNAIRL
3	LTRGKQSNGLHGDYDVE	SAHEVGVGY	PLRVFRKTTPLVNAI
4	GLQVKPLGNNSWTLEPAPAPKEDALTVV	RSGTEAHAW	VFRKTTPLVNAIRLS
5	WLGDARENDVFEHAGARDVI	KTRHTGLET	TPLRVFRKTTPLVNA
6	REDFAKT	DYKPGNWTF	
7	PENNGTGSHDL	QTNDTVTAR	
8	APYGQPQLSLAPVSL	HEVGVGYRY	
9	AVRYGPQ	RGKTRHTGL	
10	IPQDFGIEAGV	QSNGLHGDY	
11	SPTSSQNNPK	VAYDFGPQM	
12	RGSDWREHSATR	NPKETHNLM	
13	YAPDEV	FLINFNNQY	
14	YDGEADMPGGLSRADYDADRWQSTRPYDRFWGRR	HLTDSWNLY	
15	QFQPDSQHKF	DWREHSATR	
16	TLRSGYLEQGKRITLSPRN		
17	NESTHEMRYYTATSSGQLPSGSSPYDRDTRSG		
18	ESYQNNAITGTHEEVSYNA		
19	FGTVQYSQIGKAVQSGNVE		
20	QYDSNQTNDTVTA		
21	DLGTLTPTLD		
22	REKGDTYGNLVPFS		
23	KPGNWTF		
24	DFQSSQFADNANTVKESADGSTGR		
25	DFGPQMA		
26	SYNDNNKG		

### Predicted epitopes comparison for vaccine construct

**Figure 5:**
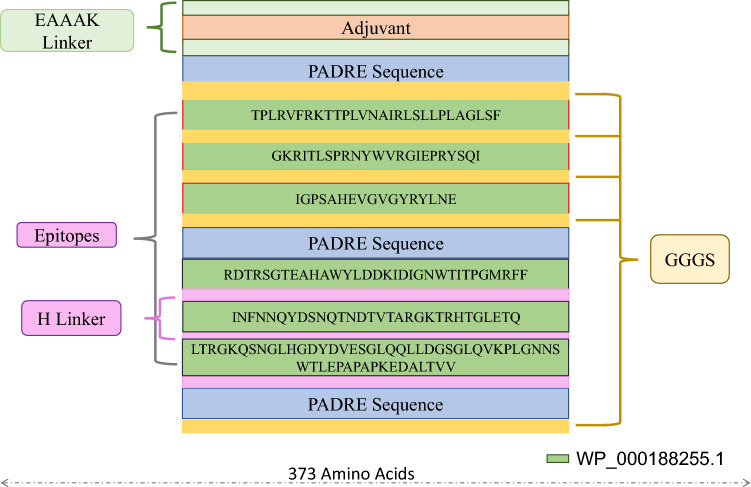
Schematic presentation of the final multi-epitope vaccine peptide. The 373 amino acid long peptide sequence containing adjuvant (brown) at both N and C terminal was linked with the multi-epitope sequence through an EAAAK linker (green). GGGS linkers (yellow) while the peptiope epitopes are linked with GGGS linkers (dark green).

The epitopes that prompt immune response to arousing both B-cell and T-cell immunity are significantly necessary for multi-epitope-based vaccine development. The predicted B-cell, MHC-I, and MHC-II epitopes were manually compared with each other to finalize the similar epitopes present in the resultant epitopes of B-cell, MHC-I and MHC-II epitopes for the construction of the final vaccine construct. These vaccines should be able to stimulate B-cell, MHC-II, and MHC-II molecules (Table 3). Finally, we shortlisted only 6 similar epitopes on the basis of similarities among the B-cell epitopes and MHC-I, MHC-II i.e., TPLRVFRKTTPLVNAIRLSLLPLAGLSF, GKRITLSPRNYWVRGIEPRYSQI, IGPSAHEVGVGYRYLNE, RDTRSGTEAHAWYLDDKIDIGNWTITPGMRFF, INFNNQYDSNQTNDTVTARGKTRHTGLETQ, and LTRGKQSNGLHGDYDVESGLQQLLDGSGLQVKPLGNNSWTLEPAPAPKEDALTVV, respectively (Fig. 5).

### Vaccine construction

During the vaccine construction, different epitope sequences were added in different combination. Different linkers such as GGGS were used between these sequences to design various combination of vaccine construct. Different combination of epitope sequences were constructed with four different adjuvants i.e., HBHA protein, beta-defensin, L7/L12 ribosomal protein, and HBHA conserved sequence, respectively to design vaccine models^61^. The use of amino acid linkers such as GGGS enhances the immunogenicity whereas PADRE (Pan HLA-DR reactive epitope) sequence helps in the induction of CD4^+^ T-cells that improve efficacy and potency of peptide vaccine^60^. Beta-defensin adjuvant is an agonist of the TLR1, TLR2, and TLR4 while adjuvant HBHA and ribosomal adjuvant sequences are agonists of the toll-like receptor 4 (TLR4). The shortlisted B-cell, MHC-I, and MHC-II epitopes were linked in a sequential manner with corresponding adjuvant, PADRE sequence, GGGS, and EAAAK linker to design the different combination of vaccine construct. Various combination of epitope sequences were constructed with four different adjuvants i.e. beta-defensin, L7/L12 ribosomal protein, HBHA protein, and HBHA conserved sequence, respectively^61^. The use of linkers boosts the immunogenicity whereas PADRE sequence helps in the initiation of CD4^+^ cells^60^. Twelve vaccines were constructed with different combination of adjuvants and linkers as shown in Table 4.

**Table 4:** Vaccine model constructs.

S. No	Vaccine construct	Vaccine composition	Sequence
1	V1	WP_00018255.1 HBHA adjuvant, (2–28, 405–428 and 64–120)	EAAAKMAENPNIDDLPAPLLAALGAADLALATVNDLIANLRERAEETRAETRTRVEERRARLTKFQEDLPEQFIELRDKFTTEELRKAAEGYLEAATNRYNELVERGEAALQRLRSQTAFEDASARAEGYVDQAVELTQEALGTVASQTRAVGERAAKLVGIELEAAAK*AKFVAAWTLKAAA*GGGSTPLRVFRKTTPLVNAIRLSLLPLAGLSFGGGSGKRITLSPRNYWVRGIEPRYSQIGGGS*AKFVAAWTLKAAA*LTRGKQSNGLHGDYDVESGLQQLLDGSGLQVKPLGNNSWTLEPAPAPKEDALTVVHEYGAEALERAG*AKFVAAWTLKAAA*GGGS
2	V2	WP_00018255.1 HBHA adjuvant, (430–447, 593–625 and 2–28)	EAAAKMAENPNIDDLPAPLLAALGAADLALATVNDLIANLRERAEETRAETRTRVEERRARLTKFQEDLPEQFIELRDKFTTEELRKAAEGYLEAATNRYNELVERGEAALQRLRSQTAFEDASARAEGYVDQAVELTQEALGTVASQTRAVGERAAKLVGIELEAAAK*AKFVAAWTLKAAA*GGGSIGPSAHEVGVGYRYLNEGGGSINFNNQYDSNQTNDTVTARGKTRHTGLETQGGGSTPLRVFRKTTPLVNAIRLSLLPLAGLSFHEYGAEALERAG*AKFVAAWTLKAAA*GGGS
3	V3	WP_00018255.1 HBHA conserved adjuvant, (2–28, 405–428, 64–120 and 593–625)	EAAAKMAENSNIDDIKAPLLAALGAADLALATVNELITNLRERAEETRRSRVEESRARLTKLQEDLPEQLTELREKFTAEELRKAAEGYLEAATSELVERGEAALERLRSQQSFEEVSARAEGYVDQAVELTQEALGTVASQVEGRAAKLVGIELEAAAK*AKFVAAWTLKAAA*GGGSTPLRVFRKTTPLVNAIRLSLLPLAGLSFGGGSGKRITLSPRNYWVRGIEPRYSQIGGGSLTRGKQSNGLHGDYDVESGLQQLLDGSGLQVKPLGNNSWTLEPAPAPKEDALTVVGGGS*AKFVAAWTLKAAA*INFNNQYDSNQTNDTVTARGKTRHTGLETQHEYGAEALERAG*AKFVAAWTLKAAA*GGGS
4	V4	WP_00018255.1 HBHA conserved adjuvant, (430–447, 593–64,64–120 and 2–28)	EAAAKMAENSNIDDIKAPLLAALGAADLALATVNELITNLRERAEETRRSRVEESRARLTKLQEDLPEQLTELREKFTAEELRKAAEGYLEAATSELVERGEAALERLRSQQSFEEVSARAEGYVDQAVELTQEALGTVASQVEGRAAKLVGIELEAAAKAKFVAAWTLKAAAGGGSIGPSAHEVGVGYRYLNEGGGSINFNNQYDSNQTNDTVTARGKTRHTGLETQGGGS*AKFVAAWTLKAAA*LTRGKQSNGLHGDYDVESGLQQLLDGSGLQVKPLGNNSWTLEPAPAPKEDALTVVHEYGAEALERAGTPLRVFRKTTPLVNAIRLSLLPLAGLSFHEYGAEALERAG*AKFVAAWTLKAAA*GGGS
5	V5	WP_00018255.1 Beta defensin adjuvant (2–28, 405–428, 64–120)	EAAAKGIINTLQKYYCRVRGGRCAVLSCLPKEEQIGKCSTRGRKCCRRKKEAAAK*AKFVAAWTLKAAA*GGGSTPLRVFRKTTPLVNAIRLSLLPLAGLSFGGGSGKRITLSPRNYWVRGIEPRYSQIGGGS*AKFVAAWTLKAAA*LTRGKQSNGLHGDYDVESGLQQLLDGSGLQVKPLGNNSWTLEPAPAPKEDALTVVHEYGAEALERAG*AKFVAAWTLKAAA*GGGS
6	V6	WP_00018255.1 Beta defensin adjuvant (430–447, 593–625, 64–120 and 2–28)	EAAAKGIINTLQKYYCRVRGGRCAVLSCLPKEEQIGKCSTRGRKCCRRKKEAAAK*AKFVAAWTLKAAA*GGGSIGPSAHEVGVGYRYLNEGGGSINFNNQYDSNQTNDTVTARGKTRHTGLETQGGGSAKFVAAWTLKAAALTRGKQSNGLHGDYDVESGLQQLLDGSGLQVKPLGNNSWTLEPAPAPKEDALTVVHEYGAEALERAGTPLRVFRKTTPLVNAIRLSLLPLAGLSFHEYGAEALERAG*AKFVAAWTLKAAA*GGGS
7	V7	WP_00018255.1 Ribosomal adjuvant (2–28, 405–428, 64–120 and 593–625)	EAAAKMAKLSTDELLDAFKEMTLLELSDFVKKFEETFEVTAAAPVAVAAAGAAPAGAAVEAAEEQSEFDVILEAAGDKKIGVIKVVREIVSGLGLKEAKDLVDGAPKPLLEKVAKEAADEAKAKLEAAGATVTVKEAAAK*AKFVAAWTLKAAA*GGGSTPLRVFRKTTPLVNAIRLSLLPLAGLSFGGGSGKRITLSPRNYWVRGIEPRYSQIGGGS*AKFVAAWTLKAAA*LTRGKQSNGLHGDYDVESGLQQLLDGSGLQVKPLGNNSWTLEPAPAPKEDALTVVHEYGAEALERAGINFNNQYDSNQTNDTVTARGKTRHTGLETQHEYGAEALERAG*AKFVAAWTLKAAA*GGGS
8	V8	WP_00018255.1 Ribosomal adjuvant (430–447, 593–625, 64–120 and 2–28)	EAAAKMAKLSTDELLDAFKEMTLLELSDFVKKFEETFEVTAAAPVAVAAAGAAPAGAAVEAAEEQSEFDVILEAAGDKKIGVIKVVREIVSGLGLKEAKDLVDGAPKPLLEKVAKEAADEAKAKLEAAGATVTVKEAAAK*AKFVAAWTLKAAA*GGGSIGPSAHEVGVGYRYLNEGGGSINFNNQYDSNQTNDTVTARGKTRHTGLETQGGGS*AKFVAAWTLKAAA*LTRGKQSNGLHGDYDVESGLQQLLDGSGLQVKPLGNNSWTLEPAPAPKEDALTVVHEYGAEALERAGTPLRVFRKTTPLVNAIRLSLLPLAGLSFHEYGAEALERAG*AKFVAAWTLKAAA*GGGS
9	V9	WP_00018255.1 HBHA adjuvant, (64–120, 430–447, 593–625 and 405–428)	EAAAKMAENPNIDDLPAPLLAALGAADLALATVNDLIANLRERAEETRAETRTRVEERRARLTKFQEDLPEQFIELRDKFTTEELRKAAEGYLEAATNRYNELVERGEAALQRLRSQTAFEDASARAEGYVDQAVELTQEALGTVASQTRAVGERAAKLVGIELEAAAK*AKFVAAWTLKAAA*GGGSLTRGKQSNGLHGDYDVESGLQQLLDGSGLQVKPLGNNSWTLEPAPAPKEDALTVVGGGSIGPSAHEVGVGYRYLNEGGGS*AKFVAAWTLKAAA*INFNNQYDSNQTNDTVTARGKTRHTGLETQHEYGAEALERAGGKRITLSPRNYWVRGIEPRYSQIHEYGAEALERAG*AKFVAAWTLKAAA*GGGS
10	V10	WP_00018255.1 HBHA conserved adjuvant, (405–428, 593–625, 64–120 and 430-44t)	EAAAKMAENSNIDDIKAPLLAALGAADLALATVNELITNLRERAEETRRSRVEESRARLTKLQEDLPEQLTELREKFTAEELRKAAEGYLEAATSELVERGEAALERLRSQQSFEEVSARAEGYVDQAVELTQEALGTVASQVEGRAAKLVGIELEAAAK*AKFVAAWTLKAAA*GGGSGKRITLSPRNYWVRGIEPRYSQIGGGSINFNNQYDSNQTNDTVTARGKTRHTGLETQGGGS*AKFVAAWTLKAAA*LTRGKQSNGLHGDYDVESGLQQLLDGSGLQVKPLGNNSWTLEPAPAPKEDALTVVHEYGAEALERAGIGPSAHEVGVGYRYLNEHEYGAEALERAG*AKFVAAWTLKAAA*GGGS
11	V11	WP_00018255.1 Beta defensin adjuvant (64–120, 2–28, 405–428 and 593–625)	EAAAKGIINTLQKYYCRVRGGRCAVLSCLPKEEQIGKCSTRGRKCCRRKKEAAAK*AKFVAAWTLKAAA*GGGSLTRGKQSNGLHGDYDVESGLQQLLDGSGLQVKPLGNNSWTLEPAPAPKEDALTVVGGGSTPLRVFRKTTPLVNAIRLSLLPLAGLSFGGGS*AKFVAAWTLKAAA*IGPSAHEVGVGYRYLNEHEYGAEALERAGINFNNQYDSNQTNDTVTARGKTRHTGLETQHEYGAEALERAG*AKFVAAWTLKAAA*GGGS
12	V12	WP_00018255.1 Ribosomal adjuvant (405–428, 593–625, 2–28 and 64–120)	EAAAKMAKLSTDELLDAFKEMTLLELSDFVKKFEETFEVTAAAPVAVAAAGAAPAGAAVEAAEEQSEFDVILEAAGDKKIGVIKVVREIVSGLGLKEAKDLVDGAPKPLLEKVAKEAADEAKAKLEAAGATVTVKEAAAK*AKFVAAWTLKAAA*GGGSIGPSAHEVGVGYRYLNEGGGSINFNNQYDSNQTNDTVTARGKTRHTGLETQGGGS*AKFVAAWTLKAAA*TPLRVFRKTTPLVNAIRLSLLPLAGLSFHEYGAEALERAGLTRGKQSNGLHGDYDVESGLQQLLDGSGLQVKPLGNNSWTLEPAPAPKEDALTVVHEYGAEALERAG*AKFVAAWTLKAAA*GGGS

### Allergenicity, antigenicity and solubility prediction

The AlgPred score higher than −0.8 was considered as allergenic. The result showed that out of twelve vaccines three were allergenic, therefore subsequently excluded while the remaining nine vaccines were assessed for solubility. The solubility of vaccine constructs was determined to express easily in the cloning of *E. coli* vector through SoLpro tool. The results showed that all vaccines are soluble as the score values are estimated more than the threshold value (0.6). The ANTIGENpro and VaxiJen tools were also used with a default threshold of 0.5 for antigenicity prediction. The result showed that seven vaccines (V3, V4, V5, V6, V9, V10, V11) have higher antigenic scores than the threshold value of 0.5 (Table 5).

**Table 5:** Allergenicity, antigenicity, solubility predicted for 12 vaccine constructs.

Vaccine	Allergenicity	Antigenicity	Solubility	Vaxijen
V1	−1.02	0.67	0.89	–
V2	−0.69	0.67	0.89	–
V3	−0.87	0.80	0.91	0.86
V4	−0.60	0.84	0.95	0.80
V5	−1.10	0.85	0.96	0.93
V6	−0.62	0.85	0.94	0.91
V7	0.02	0.82	0.93	–
V8	0.23	0.84	0.95	–
V9	−0.69	0.88	0.97	0.85
V10	−0.61	0.86	0.94	0.89
V11	−0.62	0.86	0.96	0.91
V12	0.23	0.84	0.95	–

### Physicochemical analysis of shortlisted vaccines constructs

The physicochemical properties i.e., hydropathicity index number of amino acids, aliphatic index, PI value, molecular weight, and instability index, of all seven shortlisted vaccine constructs were assessed via ProtParam server. The molecular weight was estimated to be 24–39 kDa with a pI score of 5.2–10.06. Whereas, the Instability Index (II) value was found to be stable for all shortlisted vaccine constructs. The grand average of hydropathicity was found to be (−0.2 to 0.4) to initiate an immunogenic reaction response (Supplementary Table 3).

### Structure prediction and validation

**Figure 6:**
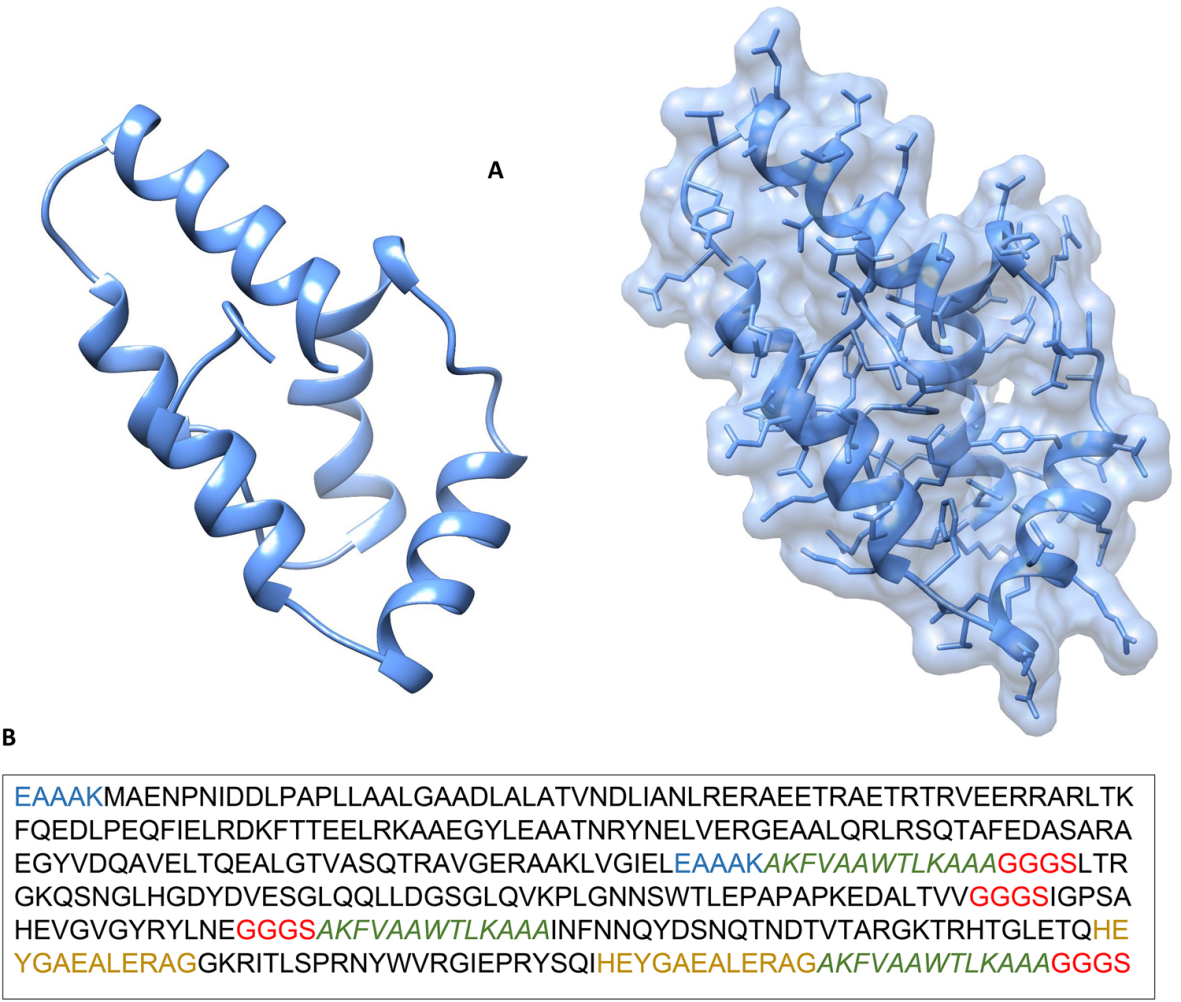
Vaccine structure modeling and Validation. (**A**) The 3D model of a multi-epitope vaccine was obtained by Swiss Model and (**B**) Vaccine sequence.

The 3D structure of shortlisted seven vaccines was modeled through Swiss-Model tool^47^. On the basis of modeled structure and template sequence similarities, V9 vaccine construct was shortlisted as final vaccine model. Selection of the model was purely based on the presence of a high percentage of residues in most favorable region of the Ramachandran plot. The template identified for V9 was lipid binding protein i.e. Ce-FAR-7 with PDB ID:2w9y (Fig. 6). In terms of stereochemical quality, the modeled structure showed that 91.1% residues lie in the most favorable region with 8.1% residues in additionally allowed region, respectively (Supplementary Fig. 3a). Similarly, PSIPRED tool was used to predict and validate the 2D molded structure of vaccine. The structure of vaccine constructs showed similar number of alpha helices and beta turns as predicted by Swiss-Model (Supplementary Fig. 3b).

### Molecular docking of vaccine construct (V9) with HLA alleles

The binding of T-cell with HLA molecules activates the adaptive immunity. A potent multi-epitope vaccine construct can initiate an immune response against several epitopes that in turn are identified in the context of different HLA allele proteins. The V9 vaccine construct was docked with the six different HLA allele proteins i.e. 3C5J (HLA-DR B3*02:02), 1H15(HLA-DR B5*01:01), 2FSE (HLA-DRB1*01:01), 2Q6W (HLA-DR B3*01:01), and 2SEB (HLA-DRB1*04:01), 1A6A (HLA-DR B1*03:01) using PatchDock and were refined through FireDock online server (Table 6).

**Table 6:** Docked score of HLA and vaccine model 9.

Vaccine construct	HLA alleles (PDB: ID)	SCORE	AREA	Hydrogen bond energy	Global energy	ACE
V9	1A6A	15,166	2152.30	−3.39	−24.48	476.15
	3C5J	14,980	2139.70	−4.29	5.95	127.27
	1H15	16,074	1995.70	−0.27	1.81	361.93
	2FSE	16,698	2234.50	0.00	4.50	462.17
	2Q6W	18,312	2524.50	0.00	6.42	443.18
	2SEB	15,958	2359.20	−0.92	−11.98	164.32
	2Z65	15,350	2469.60	0.00	−0.28	488.41

### Molecular docking of vaccine construct (V9) with TLR4/MD complex

**Figure 7:**
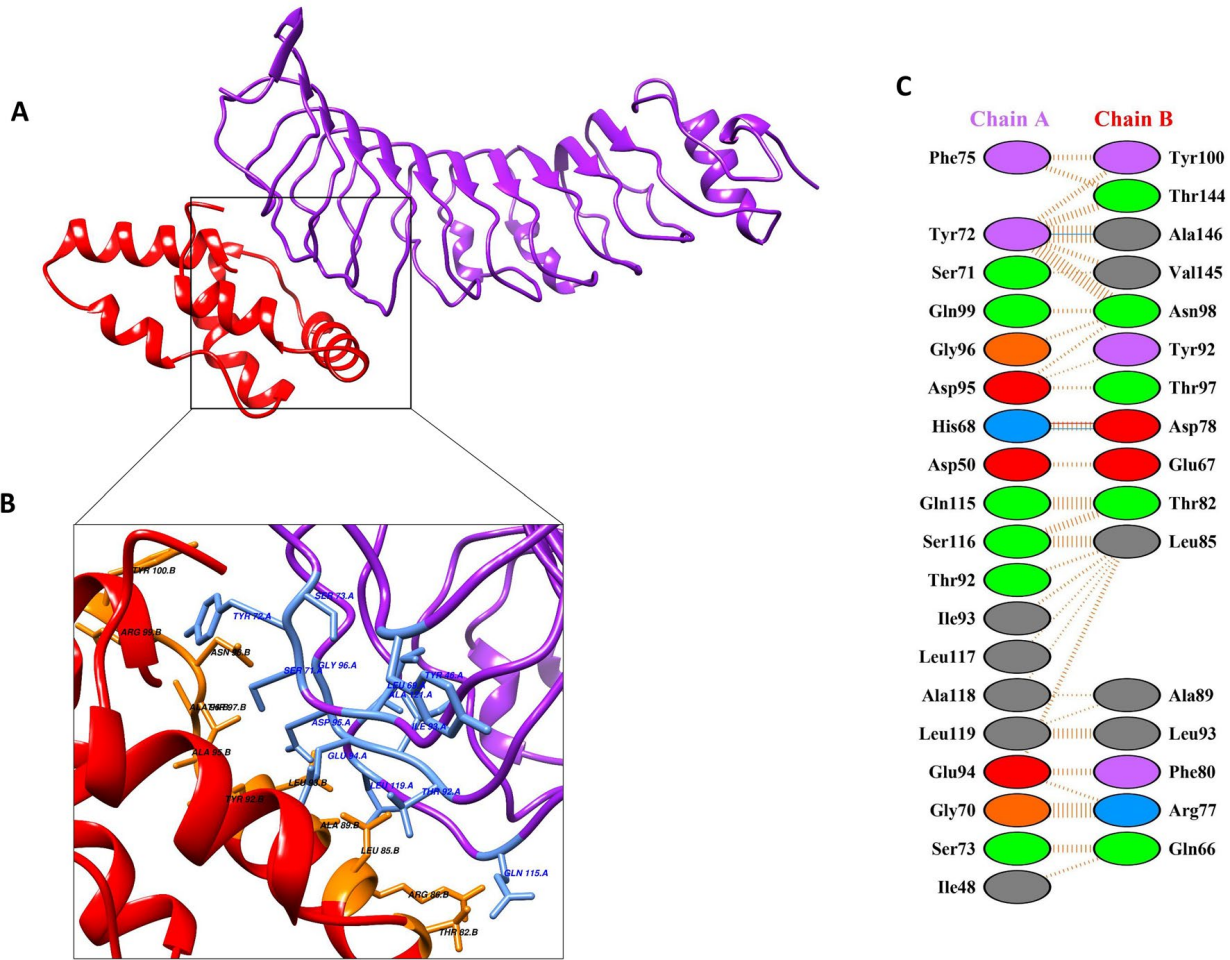
Docked vaccine construct with TLR4/MD. (**A**) Docked complex of vaccine (red) and TL4/MD (purple) (**B**), interaction occurs between the vaccine model and TLR4/MD protein. Interacting residues of vaccine represented in orange color, while protein interacting residues highlighted in blue color, (**C**) all interactions found between the docked complexes i.e., blue lines represent hydrogen bonds.

In order to boost the immune response, docking study was conducted to estimate the interactions between V9 with TLR 4/MD2 complex (PDB 2Z65) using GRAMMX tool. The V9 was comprised of the adjuvant HBHA conserved protein which acts as agonist to TLR4 protein and induced several immune responses. The PatchDock docking results in −8.9 binding energy that suggested a good interaction between V9 and TLR-4/MD2 complex. The Protein–Protein Interaction (PPIs) of vaccine 9 constructs and TLR4/MD showed that it mediates interactions with Ala146-Tyr72 and Asp78-His68 amino acids along with other interactions as highlighted in Fig. 7.

### Molecular dynamics simulation of vaccines and TLR4/MD

The molecular dynamic simulation was performed for the best docked model to validate the complex interactions and flexibility. The GROMACS simulation was performed to find the movement of molecules and atoms of vaccine constructs at 10 ns. It was observed that the complex was found to be stable at 4 ns (Supplementary Fig. 4). Furthermore, iMODs simulation analysis revealed that the Normal Mode Analysis (NMA) for the stability and mobility of vaccine-protein complex results in the deformability graph. It highlights the region of proteins having deformability illustrated in terms of the peaks, the eigenvalue of the protein and vaccine complex was found to be 1.315456e − 04. The variance association plot represents the cumulative variance of complex by green color while individual variance by red color and B-factor graph results in the clear visualization of the docked complex as shown in Supplementary Fig. 5, respectively.

### Immune response simulation

The final selected vaccine construct was used to perform a simulations study of vaccine construct under different conditions to analyze the human immune system response with C-ImmSim software^62^. The ImmSim server immune simulation outcomes confirmed consistency with real immune reactions. The C-ImmSim server resulted in the identification of B-cell, T Helper, T cytotoxic, Natural Killer cells population, interleukins productions, and Ab production. The primary response was illustrated by high IgM levels. In addition, decrease in antigenic concentration was observed and characterized as an increase in the immunoglobulin expression i.e. B-cell population, IgG1 + IgG2, IgM, and IgG + IgM. The results showed a clear increase in the population of Th (helper) and Tc (cytotoxic) cells with memory growth after the induction of V9 construct. The IFN-g production was also identified and has been stimulated after immunization as shown in Supplementary Fig. 6.

### Codon optimization and in silico cloning

**Figure 8:**
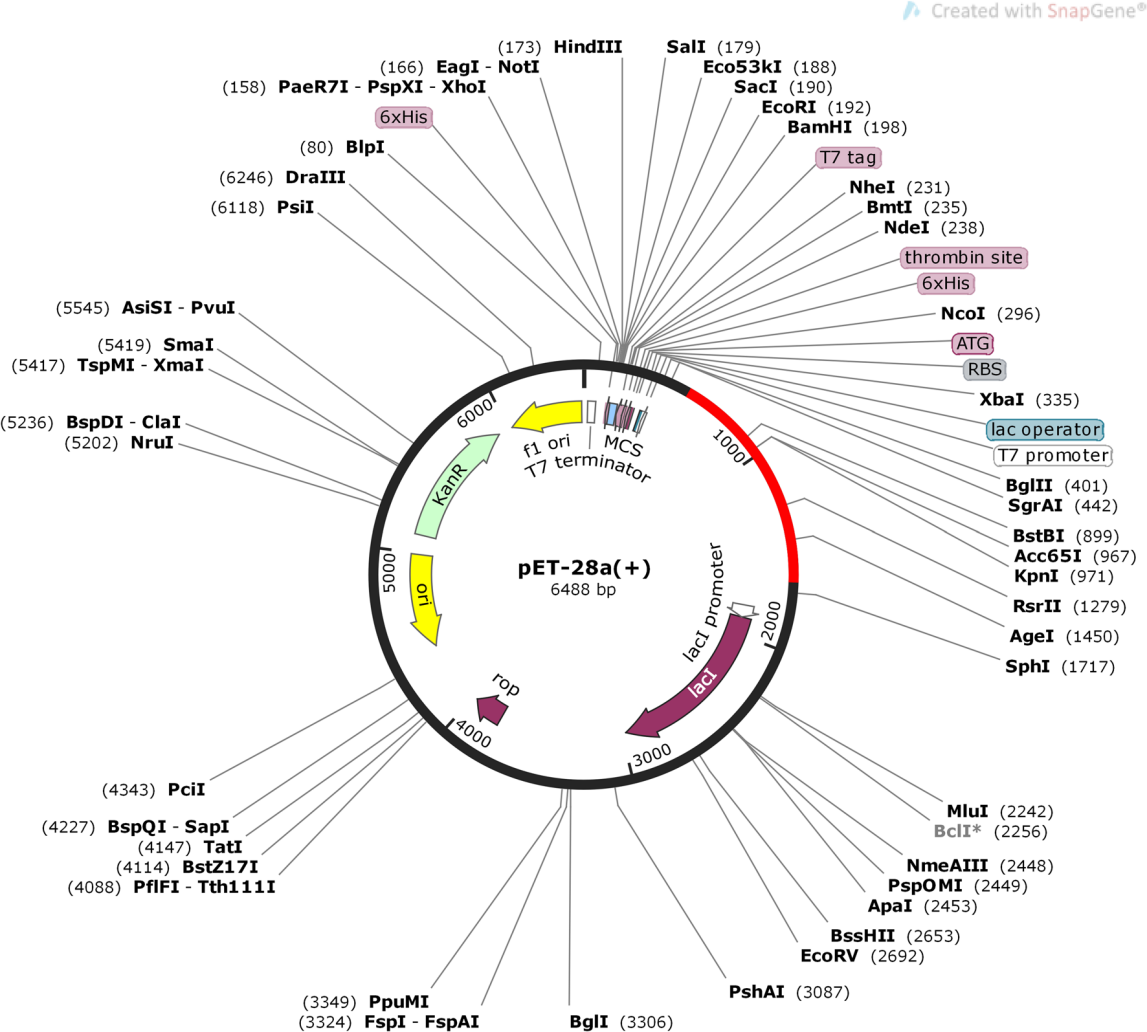
Codon optimization and in-silico cloning of vaccine model. In silico restriction cloning of the multi-epitope vaccine sequence into the pET30a ( +) expression vector using SnapGene software, the red part represents the vaccine’s gene coding, and the black circle represents the vector backbone.

The JCAT tool was used for the codon optimization and cloning of vaccine V9. The vaccine V9 was reverse translated for best expression in *E. coli* (strain K12). The average GC content and Codon Optimization Index (CAI) value for V9 was predicted to be 54.2% and 0.98, respectively, resulting in the successful expression of vaccine construct in *E. coli* system. Finally, SnapGene tool was used to introduce the adapted codon sequence (V9) to construct the recombinant plasmid into the pET30a ( +) vector (Fig. 8).

## Discussion

*Shigella dysenteriae*, an intracellular pathogen responsible for Shigellosis (bacillary dysentery) continues to be the leading cause of high mortality rate i.e., up to ~ 90 million cases annually. It is difficult to produce a safe and efficacious vaccine against *Shigella dysenteriae* due to the possibilities of *Shigella spp*. occasional back mutations. Due to the emergence of multiple drug resistant *Shigella spp*. and lack of vaccines necessitated alternative strategies to develop potential vaccine candidates against this. The advancement in Bioinformatics made it possible to predict new therapeutics (such as vaccines) with an appreciable accuracy without the essential need of living cells within less time, cost, and labor. Therefore, in the current study, a Reverse Vaccinology approach was adapted to identify unique vaccine candidates and design the novel vaccine construct against the Sd197 strain of *Shigella dysenteriae*.

In the current study, 96 proteins are identified as therapeutic drug targets through a subtractive genomic analysis approach that are uniquely present in *Shigella dysenteriae*. The subcellular localization analysis is one of the important steps to construct the vaccine candidates. The current study identified 77 proteins as essential, virulent, druggable, and cytoplasmic proteins that may act as potent drug targets against *S. dysenteriae*, while one protein was classified as outer membrane i.e. WP_000188255.1 (Fe (3+) dicitrate transport protein FecA). The outer membrane proteins play important roles in bacterial pathogenesis such as adhesion, invasion, biofilm formation, effector secretion, and cell-to-cell dissemination and thus was used as a vaccine candidate for downstream analysis. Furthermore, the antigenicity analysis showed that WP_000188255.1 is significantly antigenic. The T and B cells help to activate the immune response against foreign particles. Currently, synthetic peptides or epitopes are used to produce this immune response. Herein, the identified MHC-I-II and B-Cells epitopes were predicted from WP_000188255.1 protein. Finally, vaccine models were constructed against *S. dysenteriae* using different combination of shortlisted epitopes along with four different adjuvants. Based on toxicity, immunogenicity, conservancy, pattern of allergenicity, physio-chemical properties, structural stability, and structure stereochemistry criteria, only V9 construct was found to be the most favorable vaccine construct. Furthermore, the interaction of modeled vaccine construct with Human Leukocyte Antigen (HLA) to elucidate effective immune response was studied using molecular docking simulation studies. Furthermore, molecular docking and simulation studies, immune simulation studies, and codon optimization confirmed the complex stability (vaccine and HLAs) and its binding affinity, induction of immune cells response against clearance of antigen and provided optimistic CAI value that helps in *in-vivo* expression (J. Li et al., 2021).

Eventually, to the best of our knowledge, the proposed peptides against *S. dysenteriae* are the potent and unique epitopes identified through computational approaches. The number of significant proteins highlighted in this study put forward a pipeline for the identification and designing of a vaccine model to generate an immune response against *S. dysenteriae*. The shortlisted V9 model and WP_000188255.1 protein may be utilized as a precautionary measure before traveling and admission of patient in intensive care unit to avoid the high infection as well as mortality and morbidity rate caused by shigellosis. However, the in vivo expression and evaluation of V9 multiepitope chimeric vaccine is needed to validate the stimulation of robust immune response against *Shigella dysenteriae*. The same approach was applied by *Bazhan *et al*.*, for T-cell multiepitope vaccine modeling against *Ebola virus* that showed significant immunogenicity in mice^63^. Additionally, a similar approach has been widely applied against *Brucella*^64^, *Leishmania*^65^, *Streptococcus pneumoniae*^66^, and *Acinetobacter baumannii*^23^*,* etc. Importantly, the current user-friendly vaccine identification pipeline can be extended to other target pathogens to pave a better way for the control of transmission of persistent infection. Importantly, the proposed vaccine model V9 needs to be validated through experimental studies to ensure its safe use against *S. dysenteriae*. Since our predicted study applies all significant criteria of conservancy and essentiality, the prioritized vaccine can be widely used against all *S. dysenteriae* serotypes.

## Conclusion

Precisely, the current study applied the integrated immunoinformatics analysis based on subtractive genomic and Reverse Vaccinology approach to identify potent drug targets and design of the chimeric vaccine. Identified outer membrane protein FecA was used as a novel vaccine candidate to develop chimeric vaccine novel against *S. dysenteriae*. The designing of an effective and affordable vaccine is of great need against *Shigella dysenteriae* to reduce the global health problem. The current study highlights valuable proteomics information about the *Shigella dysenteriae* and develops a new chimeric vaccine construct that simply cannot be acquired from traditional methods. However, further in vitro, animal studies and pre-clinical analysis are suggested to be performed for the validation of our predicted vaccine model as either DNA vaccines, or as recombinant proteins for management of shigellosis.

## Supplementary Information

Supplementary Information.

## References

[1] Killackey SA, Sorbara MT, Girardin SE (2016). Cellular aspects of Shigella pathogenesis: Focus on the manipulation of host cell processes. Front. Cell. Infect. Microbiol..

[2] Kotloff KL (2013). Burden and aetiology of diarrhoeal disease in infants and young children in developing countries (the Global Enteric Multicenter Study, GEMS): A prospective, case-control study. The Lancet.

[3] Halimeh FB (2021). Historical, current, and emerging tools for identification and serotyping of Shigella. Brazil. J. Microbiol..

[4] Kotloff KL (1999). Global burden of Shigella infections: Implications for vaccine development and implementation of control strategies. Bull. World Health Organ..

[5] Mani S, Wierzba T, Walker RI (2016). Status of vaccine research and development for Shigella. Vaccine.

[6] Valério E, Chaves S, Tenreiro R (2010). Diversity and impact of prokaryotic toxins on aquatic environments: A review. Toxins.

[7] Von Seidlein L (2006). A multicentre study of Shigella diarrhoea in six Asian countries: disease burden, clinical manifestations, and microbiology. PLoS Med..

[8] Ashkenazi S, Cohen D (2013). An update on vaccines against Shigella. Therapeut. Adv. Vaccines.

[9] Nezafat N (2016). Designing an efficient multi-epitope peptide vaccine against Vibrio cholerae via combined immunoinformatics and protein interaction based approaches. Comput. Biol. Chem..

[10] Hasan M (2019). Reverse vaccinology approach to design a novel multi-epitope subunit vaccine against avian influenza A (H7N9) virus. Microb. Pathog..

[11] Zhu H (2020). Big data and artificial intelligence modeling for drug discovery. Annu. Rev. Pharmacol. Toxicol..

[12] Bambini S, Rappuoli R (2009). The use of genomics in microbial vaccine development. Drug Discovery Today.

[13] Medeiros PHQ (2020). A bivalent vaccine confers immunogenicity and protection against Shigella flexneri and enterotoxigenic Escherichia coli infections in mice. NPJ Vaccines.

[14] Kanehisa M, Goto S (2000). KEGG: kyoto encyclopedia of genes and genomes. Nucl. Acids Res..

[15] Tatusova TA, Karsch-Mizrachi I, Ostell JA (1999). Complete genomes in WWW Entrez: data representation and analysis. Bioinform. (Oxford, England).

[16] UniProt: the universal protein knowledgebase in 2021. *Nucl. Acids Res.* **49**, D480-D489 (2021). 10.1093/nar/gkaa1100 PMC7778908 33237286

[17] Li W, Godzik A (2006). Cd-hit: a fast program for clustering and comparing large sets of protein or nucleotide sequences. Bioinformatics.

[18] Zhang R, Ou HY, Zhang CT (2004). DEG: a database of essential genes. Nucl. Acids Res..

[19] Wishart, D. S. *et al.* DrugBank 5.0: a major update to the DrugBank database for 2018. *Nucleic acids research* **46**, D1074-D1082 (2018). 10.1093/nar/gkx1037 PMC5753335 29126136

[20] Kanehisa M, Furumichi M, Sato Y, Ishiguro-Watanabe M, Tanabe M (2021). KEGG: integrating viruses and cellular organisms. Nucl. Acids Res..

[21] Moriya Y, Itoh M, Okuda S, Yoshizawa AC, Kanehisa M (2007). KAAS: An automatic genome annotation and pathway reconstruction server. Nucleic Acids Res..

[22] Liu B, Zheng D, Jin Q, Chen L, Yang J (2019). VFDB 2019: A comparative pathogenomic platform with an interactive web interface. Nucleic Acids Res..

[23] Solanki V, Tiwari V (2018). Subtractive proteomics to identify novel drug targets and reverse vaccinology for the development of chimeric vaccine against Acinetobacter baumannii. Sci. Rep..

[24] Gupta SK (2014). ARG-ANNOT, a new bioinformatic tool to discover antibiotic resistance genes in bacterial genomes. Antimicrob. Agents Chemother..

[25] Yu, N. Y. *et al.* PSORTb 3.0: improved protein subcellular localization prediction with refined localization subcategories and predictive capabilities for all prokaryotes. *Bioinformatics* **26**, 1608–1615 (2010). 10.1093/bioinformatics/btq249 PMC2887053 20472543

[26] Doytchinova IA, Flower DR (2007). VaxiJen: A server for prediction of protective antigens, tumour antigens and subunit vaccines. BMC Bioinformatics.

[27] Doytchinova IA, Guan P, Flower DR (2006). EpiJen: A server for multistep T cell epitope prediction. BMC Bioinform..

[28] Ni Z, Chen Y, Ong E, He Y (2017). Antibiotic resistance determinant-focused Acinetobacter baumannii vaccine designed using reverse vaccinology. Int. J. Mol. Sci..

[29] Kim Y (2012). Immune epitope database analysis resource. Nucleic Acids Res..

[30] Calis, J. J. *et al.* Properties of MHC class I presented peptides that enhance immunogenicity. *PLoS Comput. Biol.* **9**, e1003266 (2013). 10.1371/journal.pcbi.1003266 PMC3808449 24204222

[31] Bui H-H, Sidney J, Li W, Fusseder N, Sette A (2007). Development of an epitope conservancy analysis tool to facilitate the design of epitope-based diagnostics and vaccines. BMC Bioinformatics.

[32] Karthik, L. *et al.* Protease inhibitors from marine actinobacteria as a potential source for antimalarial compound. *PloS One* **9**, e90972 (2014). 10.1371/journal.pone.0090972 PMC3949715 24618707

[33] Webb, B. & Sali, A. Comparative protein structure modeling using MODELLER. *Curr. Protoc. Bioinform.* **54**, 5.6. 1–5.6. 37 (2016). 10.1002/cpbi.3 PMC5031415 27322406

[34] Anand Y, Pande S, Gore D (2013). Reverse vaccinology: An approach to search vaccine leads of Shigella sonnei. J. Pharm. Res..

[35] Bhattacharya M (2020). Computational characterization of epitopic region within the outer membrane protein candidate in Flavobacterium columnare for vaccine development. J. Biomol. Struct. Dyn..

[36] El-Manzalawy, Y., Dobbs, D. & Honavar, V. in *Computational Systems Bioinformatics: (Volume 7)* 121–132 (World Scientific, 2008). PMC3400678 19642274

[37] Saha, S. & Raghava, G. P. S. Prediction of continuous B‐cell epitopes in an antigen using recurrent neural network. *Prot.: Struct. Funct. Bioinform.* **65**, 40–48 (2006). 10.1002/prot.21078 16894596

[38] Barh D, Misra AN, Kumar A, Vasco A (2010). A novel strategy of epitope design in Neisseria gonorrhoeae. Bioinformation.

[39] Ponomarenko J (2008). ElliPro: A new structure-based tool for the prediction of antibody epitopes. BMC Bioinform..

[40] Emini EA, Hughes JV, Perlow D, Boger J (1985). Induction of hepatitis A virus-neutralizing antibody by a virus-specific synthetic peptide. J. Virol..

[41] Ponomarenko JV, Bourne PE (2007). Antibody-protein interactions: benchmark datasets and prediction tools evaluation. BMC Struct. Biol..

[42] Chou P, Fasman GD (2009). Amino acid sequence. Adv. Enzymol. Relat. Areas Mol. Biol..

[43] Yukeswaran, L., Shreeranjana, S. & Subhashini, T. Immunoinformatics Aided Multi-epitope Based Vaccine Design Against Crimean-Congo Virus. *AIJR Abst.*, 43 (2021).

[44] ul Qamar, M. T. *et al.* Designing multi-epitope vaccine against Staphylococcus aureus by employing subtractive proteomics, reverse vaccinology and immuno-informatics approaches. *Comput. Biol. Med.* **132**, 104389 (2021). 10.1016/j.compbiomed.2021.104389 33866250

[45] Gasteiger, E. *et al.* Protein identification and analysis tools on the ExPASy server. *Proteom. Protoc. Hand.*, 571–607 (2005). 10.1385/1-59259-584-7:531 10027275

[46] Wang, W. *et al.* Identification of Vibrio parahaemolyticus and Vibrio Spp. specific outer membrane proteins by reverse vaccinology and surface proteome. *Front. Microbiol.* **11**, 3529 (2020). 10.3389/fmicb.2020.625315 PMC7901925 33633699

[47] Waterhouse A (2018). SWISS-MODEL: Homology modelling of protein structures and complexes. Nucl. Acids Res..

[48] Bienert S (2017). The SWISS-MODEL Repository—new features and functionality. Nucl. Acids Res..

[49] McGuffin LJ, Bryson K, Jones DT (2000). The PSIPRED protein structure prediction server. Bioinformatics.

[50] Laskowski RA, MacArthur MW, Moss DS, Thornton JM (1993). PROCHECK: A program to check the stereochemical quality of protein structures. J. Appl. Crystallogr..

[51] Mashiach E, Schneidman-Duhovny D, Andrusier N, Nussinov R, Wolfson HJ (2008). FireDock: A web server for fast interaction refinement in molecular docking. Nucl. Acids Res..

[52] Tovchigrechko A, Vakser IA (2006). GRAMM-X public web server for protein–protein docking. Nucl. Acids Res..

[53] Pettersen EF (2004). UCSF Chimera—a visualization system for exploratory research and analysis. J. Comput. Chem..

[54] Laskowski RA (2001). PDBsum: summaries and analyses of PDB structures. Nucl. Acids Res..

[55] Tiwari V, Tiwari M, Biswas D (2018). Rationale and design of an inhibitor of RecA protein as an inhibitor of Acinetobacter baumannii. J. Antibiot..

[56] Rahman N (2020). Vaccine design from the ensemble of surface glycoprotein epitopes of SARS-CoV-2: An immunoinformatics approach. Vaccines.

[57] Grote A (2005). JCat: A novel tool to adapt codon usage of a target gene to its potential expression host. Nucl. Acids Res..

[58] Braun V, Mahren S, Ogierman M (2003). Regulation of the FecI-type ECF sigma factor by transmembrane signalling. Curr. Opin. Microbiol..

[59] Moxon R, Reche PA, Rappuoli R (2019). reverse vaccinology. Front. Immunol..

[60] Ghaffari-Nazari, H. *et al.* Improving multi-epitope long peptide vaccine potency by using a strategy that enhances CD4+ T help in BALB/c mice. *PloS one* **10**, e0142563 (2015). 10.1371/journal.pone.0142563 PMC4640540 26556756

[61] Yang Y (2015). In silico design of a DNA-based HIV-1 multi-epitope vaccine for Chinese populations. Hum. Vaccin. Immunother..

[62] Puzone R, Kohler B, Seiden P, Celada F (2002). IMMSIM, a flexible model for in machina experiments on immune system responses. Futur. Gener. Comput. Syst..

[63] Bazhan SI (2019). In silico designed ebola virus T-cell multi-epitope DNA vaccine constructions are immunogenic in mice. Vaccines.

[64] Hisham, Y. & Ashhab, Y. Identification of cross-protective potential antigens against pathogenic Brucella spp. through combining pan-genome analysis with reverse vaccinology. *J. Immunol. Res.* **2018** (2018). 10.1155/2018/1474517 PMC6304850 30622973

[65] John L, John GJ, Kholia T (2012). A reverse vaccinology approach for the identification of potential vaccine candidates from Leishmania spp. Appl. Biochem. Biotechnol..

[66] Talukdar S, Zutshi S, Prashanth K, Saikia KK, Kumar P (2014). Identification of potential vaccine candidates against Streptococcus pneumoniae by reverse vaccinology approach. Appl. Biochem. Biotechnol..

