# Supplementary material for: Identification of vaccine and drug targets in Shigella dysenteriae sd197 using reverse vaccinology approach
Source: Sci Rep. 2022 Jan 7;12:251. doi: 10.1038/s41598-021-03988-0 (PMC8742002; doi:10.1038/s41598-021-03988-0)
Supplement: Supplementary file 1 — Supplementary Information. [file 41598_2021_3988_MOESM1_ESM.docx]

| Supplementary Table 1: Identified Potent Drug Targets and Vaccine Candidates against *S. dysenteriae* | | | |
| --- | --- | --- | --- |
| S. No. | Drug Targets IDs | Drug Targets Names | Drug Compounds IDs from FDA library |
| 1 | WP_000010706.1 | nitric oxide reductase transcriptional regulator NorR | DB01857 |
| 2 | WP_000019351.1 | superoxide response transcriptional regulator SoxS | DB03142, DB04062 |
| 3 | WP_000024914.1 | multidrug efflux RND transporter permease subunit | DB03825, DB04209, DB07690 |
| 4 | WP_000044393.1 | MULTISPECIES: glycine cleavage system transcriptional regulator GcvA | DB03793 |
| 5 | WP_000070491.1 | MULTISPECIES: two-component system response regulator NarL | DB02355; DB02596; DB07706 |
| 6 | WP_000090187.1 | glutathione ABC transporter substrate-binding protein GsiB | DB03374 |
| 7 | WP_000101737.1 | MULTISPECIES: multidrug efflux transporter transcriptional repressor AcrR | DB03467; DB04216; DB07810 |
| 8 | WP_000104211.1 | MULTISPECIES: transcriptional regulator TdcA | DB02355, DB02596; DB07706 |
| 9 | WP_000113934.1 | phosphate response regulator transcription factor PhoB | DB02355; DB02596; DB07706 |
| 10 | WP_000137877.1 | MULTISPECIES: two-component | DB02355; DB02596; DB07706 |
| 11 | WP_000148492.1 | sigma-54-dependent response regulator transcription factor ZraR | DB01857, DB02461, DB01972 |
| 12 | WP_000171277.1 | TonB system transport protein TonB | DB02767; DB04147; DB08231 |
| 13 | WP_000179522.1 | mal regulon transcriptional regulator MalI | DB02283, DB01862; DB08297 |
| 14 | WP_000186086.1 | two-component system response regulator KdpE | DB02355; DB02596; DB07706 |
| 15 | WP_000188255.1 | MULTISPECIES: Fe(3+) dicitrate transport protein FecA | DB04039, DB01814, |
| 16 | WP_000190986.1 | HTH-type transcriptional repressor PurR | DB02283, DB01862; DB08297 |
| 17 | WP_000193447.1 | MULTISPECIES: two-component | DB01857, DB02461, DB03487 |
| 18 | WP_000201064.1 | MULTISPECIES: HTH-type transcriptional regulator GalR | DB02283, DB01862; DB08297 |
| 19 | WP_000212445.1 | MULTISPECIES: transcriptional regulator EbgR | DB02283, DB01862; DB08297 |
| 20 | WP_000217372.1 | HTH-type transcriptional activator RhaS | DB03142 |
| 21 | WP_000226408.1 | Glyoxylate bypass operon transcriptional repressor IclR | DB01942 |
| 22 | WP_000241239.1 | 4-hydroxythreonine-4-phosphate dehydrogenase PdxA | DB02609 |
| 23 | WP_000249362.1 | type II secretion system ATPase GspE | DB04395, DB02930 |
| 24 | WP_000331370.1 | formate dehydrogenase O subunit beta | DB08689 |
| 25 | WP_000365790.1 | MULTISPECIES: HTH-type transcriptional activator IlvY | DB03793 |
| 26 | WP_000366509.1 | LysR family transcriptional | DB03793 |
| 27 | WP_000371661.1 | MULTISPECIES: MDR efflux pump AcrAB transcriptional activator RobA | DB03142, |
| 28 | WP_000379246.1 | MULTISPECIES: HTH-type transcriptional regulator HdfR | DB03793 |
| 29 | WP_000440320.1 | HTH-type transcriptional activator AaeR | DB03793 |
| 30 | WP_000443496.1 | MULTISPECIES: hydroxycarboxylate dehydrogenase HcXB | DB01694 |
| 31 | WP_000460148.1 | MULTISPECIE | DB03793 |
| 32 | WP_000492802.1 | multidrug efflux RND transporter permease | DB03825 |
| 33 | WP_000494478.1 | DNA-binding transcriptional regulator XylR | DB03142 |
| 34 | WP_000548283.1 | LysR family transcriptional regulator | DB03793 |
| 35 | WP_000573624.1 | HTH-type transcriptional regulator MetR | DB03793 |
| 36 | WP_000598644.1 | two-component system response regulator BtsR | DB02355; DB02596; DB07706 |
| 37 | WP_000611283.1 | MULTISPECIES: two-component system response regulator DcuR | DB01857 |
| 38 | WP_000611328.1 | MULTISPECIES: UvrY/SirA/GacA family response regulator transcription factor | DB02461; DB03487 |
| 39 | WP_000621916.1 | response regulator transcription factor | DB04077 |
| 40 | WP_000622272.1 | transcriptional regulator LrhA | DB03793 |
| 41 | WP_000628676.1 | HTH-type transcriptional regulator GalS | DB01862; DB08297 |
| 42 | WP_000644910.1 | MULTISPECIES: DNA-binding transcriptional regulator CytR | DB02283 |
| 43 | WP_000648592.1 | LysR family transcriptional regulator | DB03793 |
| 44 | WP_000683023.1 | murein tripeptide ABC transporter substrate-binding protein MppA | DB03374 |
| 45 | WP_000730247.1 | MULTISPECIES: gluconate operon transcriptional repressor GntR | DB01862; DB08297 |
| 46 | WP_000741827.1 | MULTISPECIES: DNA-binding transcriptional regulator LysR | DB03793 |
| 47 | WP_000776240.1 | HTH-type transcriptional regulator CysB | DB03793 |
| 48 | WP_000805930.1 | response regulator transcription factor | DB01972 |
| 49 | WP_000817681.1 | DNA-binding transcriptional regulator PgrR | DB03793 |
| 50 | WP_000852837.1 | phage shock protein operon transcriptional activator | DB01857 |
| 51 | WP_000907050.1 | HTH-type transcriptional regulator MalT | DB03793 |
| 52 | WP_000940963.1 | hybrid sensor histidine kinase/response regulator | DB02671 |
| 53 | WP_000953378.1 | nickel ABC transporter, nickel/metallophore periplasmic binding protein | DB03374 |
| 54 | WP_001011001.1 | HTH-type transcriptional regulator Cbl | DB03793 |
| 55 | WP_001011469.1 | nitrogen assimilation transcriptional regulator NAC | DB03793 |
| 56 | WP_001019523.1 | LysR family transcriptional regulator | DB03793 |
| 57 | WP_001025939.1 | MULTISPECIES: DNA-binding transcriptional regulator OxyR | DB03793 |
| 58 | WP_001033722.1 | MULTISPECIES: envelope stress response regulator transcription factor CpxR | DB02355; DB02596; DB07706 |
| 59 | WP_001036964.1 | MULTISPECIES: galactose/glucose ABC transporter substrate-binding protein MglB | DB02379 |
| 60 | WP_001041017.1 | DNA-binding transcriptional regulator YhaJ | DB03793 |
| 61 | WP_001081792.1 | multidrug transporter subunit MdtE | DB04077 |
| 62 | WP_001081872.1 | hydrogenase 2 operon protein HybA | DB08689 |
| 63 | WP_001092515.1 | two-component system response regulator RstA | DB01972, DB01857 |
| 64 | WP_001113637.1 | MULTISPECIES: nitrate/nitrite response regulator protein NarP | DB02461; DB03487 |
| 65 | WP_001120104.1 | two-component system response regulator TorR | DB01972, DB02355; DB02596; DB07706 |
| 67 | WP_001132497.1 | multidrug efflux RND transporter permease subunit | DB03825 |
| 68 | WP_001157751.1 | MULTISPECIES: two-component | DB02355; DB02596; DB07706 |
| 69 | WP_001181327.1 | MULTISPECIES: HTH-type transcriptional regulator TreR | DB01862; DB08297 |
| 70 | WP_001188664.1 | MULTISPECIES: two-component | DB02355; DB02596; DB07706 |
| 71 | WP_001194358.1 | MULTISPECIES: two-component | DB02355; DB02596; DB07706 |
| 72 | WP_001221504.1 | two-component system response regulator QseB | DB01972, DB01857 |
| 73 | WP_001222888.1 | MULTISPECIES: dipeptide ABC transporter substrate-binding protein DppA | DB03374 |
| 74 | WP_001240591.1 | formate dehydrogenase N subunit beta | DB08689 |
| 75 | WP_001250231.1 | peptide ABC transporter substrate-binding protein SapA | DB03374 |
| 76 | WP_001262180.1 | DNA-binding transcriptional regulator KdgR | DB01942 |
| 77 | WP_001265481.1 | MULTISPECIES: two-component | DB02355; DB02596; DB07706 |
| 78 | WP_001273124.1 | multidrug efflux RND transporter permease AcrD | DB03825 |
| 79 | WP_001295587.1 | MULTISPECIES: transcriptional regulator TyrR | DB01857 |
| 80 | WP_005016525.1 | MULTISPECIES: transcriptional regulator UhpA | DB04077 |
| 81 | WP_005017147.1 | LysR family transcriptional regulator | DB03793 |
| 82 | WP_005017348.1 | two-component system response regulator GlrR | DB01857 |
| 83 | WP_005017758.1 | helix-turn-helix transcriptional regulator | DB03142, DB04062 |
| 84 | WP_005017788.1 | LacI family DNA-binding transcriptional regulator | DB03142 |
| 85 | WP_005019219.1 | 4Fe-4S binding protein | DB08689 |
| 86 | WP_005019446.1 | multidrug efflux RND transporter periplasmic adaptor subunit AcrA | DB04077 |
| 87 | WP_005020332.1 | HTH-type transcriptional regulator RutR | DB08167 |
| 88 | WP_005020386.1 | response regulator transcription factor HprR | DB01972 |
| 89 | WP_005022591.1 | DNA-binding transcriptional regulator DmlR | DB03793 |
| 90 | WP_005023475.1 | oligopeptide ABC transporter substrate-binding protein OppA | DB03374 |
| 91 | WP_011378570.1 | DNA-binding transcriptional repressor LacI | DB01862; DB08297 |
| 92 | WP_011378868.1 | ABC transporter substrate-binding protein | DB03374 |
| 93 | WP_011378918.1 | ribose operon transcriptional repressor RbsR | DB02283 |
| 94 | WP_024259404.1 | hydrogenase 4 subunit HyfA | DB08689 |
| 95 | WP_024259441.1 | DNA-binding transcriptional regulator AscG | DB02283 |
| 96 | WP_024259509.1 | nitric oxide reductase transcriptional regulator NorR |  |

| Supplementary Table 1: Toxicity, Conservancy, and Antigenicity identified for MHC-I epitopes | | | | |
| --- | --- | --- | --- | --- |
| S. No | MHC-I Epitopes | Antigenicity | Toxicity | Conservancy |
| 1 | WVRGIEPRY | 1.486 | Non-Toxic | 100% |
| 2 | MRFEHIESY | 0.814 | Non-Toxic | 100% |
| 3 | SAHEVGVGY | 1.287 | Non-Toxic | 100% |
| 4 | RSGTEAHAW | 1.340 | Non-Toxic | 100% |
| 5 | KTRHTGLET | 1.177 | Non-Toxic | 100% |
| 6 | DYKPGNWTF | 0.933 | Non-Toxic | 100% |
| 7 | QTNDTVTAR | 1.922 | Non-Toxic | 100% |
| 8 | HEVGVGYRY | 1.444 | Non-Toxic | 100% |
| 9 | RGKTRHTGL | 1.104 | Non-Toxic | 100% |
| 10 | QSNGLHGDY | 01.28 | Non-Toxic | 100% |
| 11 | VAYDFGPQM | 0.981 | Non-Toxic | 100% |
| 12 | NPKETHNLM | 0.934 | Non-Toxic | 100% |
| 13 | FLINFNNQY | 0.880 | Non-Toxic | 100% |
| 14 | HLTDSWNLY | 0.941 | Non-Toxic | 100% |
| 15 | DWREHSATR | 1.035 | Non-Toxic | 100% |

| Supplementary Table 2: Physicochemical Properties of shortlisted vaccine model | | | | | | | | | |
| --- | --- | --- | --- | --- | --- | --- | --- | --- | --- |
| S.No | Vaccine | MW | PI | NO of residue | no of +ive AA | no of -ive AA | ext coeffecient | stability index | hydrophobicity |
| 1 | V3 | 39068.82 | 5.9 | 367 | 44 | 47 | 37930 | 38.23 | -0.349 |
| 2 | V4 | 39165.75 | 5.28 | 369 | 41 | 51 | 33920 | 33.05 | -0.332 |
| 3 | V5 | 24054.77 | 10.06 | 228 | 35 | 15 | 36815 | 34.47 | -0.213 |
| 4 | V6 | 27816.53 | 9.56 | 264 | 35 | 22 | 33920 | 22.67 | -0.339 |
| 6 | V9 | 39979.42 | 5.44 | 373 | 44 | 52 | 43890 | 31.67 | -0.473 |
| 7 | V10 | 38861.2 | 5.29 | 364 | 42 | 52 | 42400 | 34.97 | -0.445 |
| 8 | V11 | 27816.53 | 9.56 | 264 | 35 | 22 | 34295 | 22.06 | -0.339 |


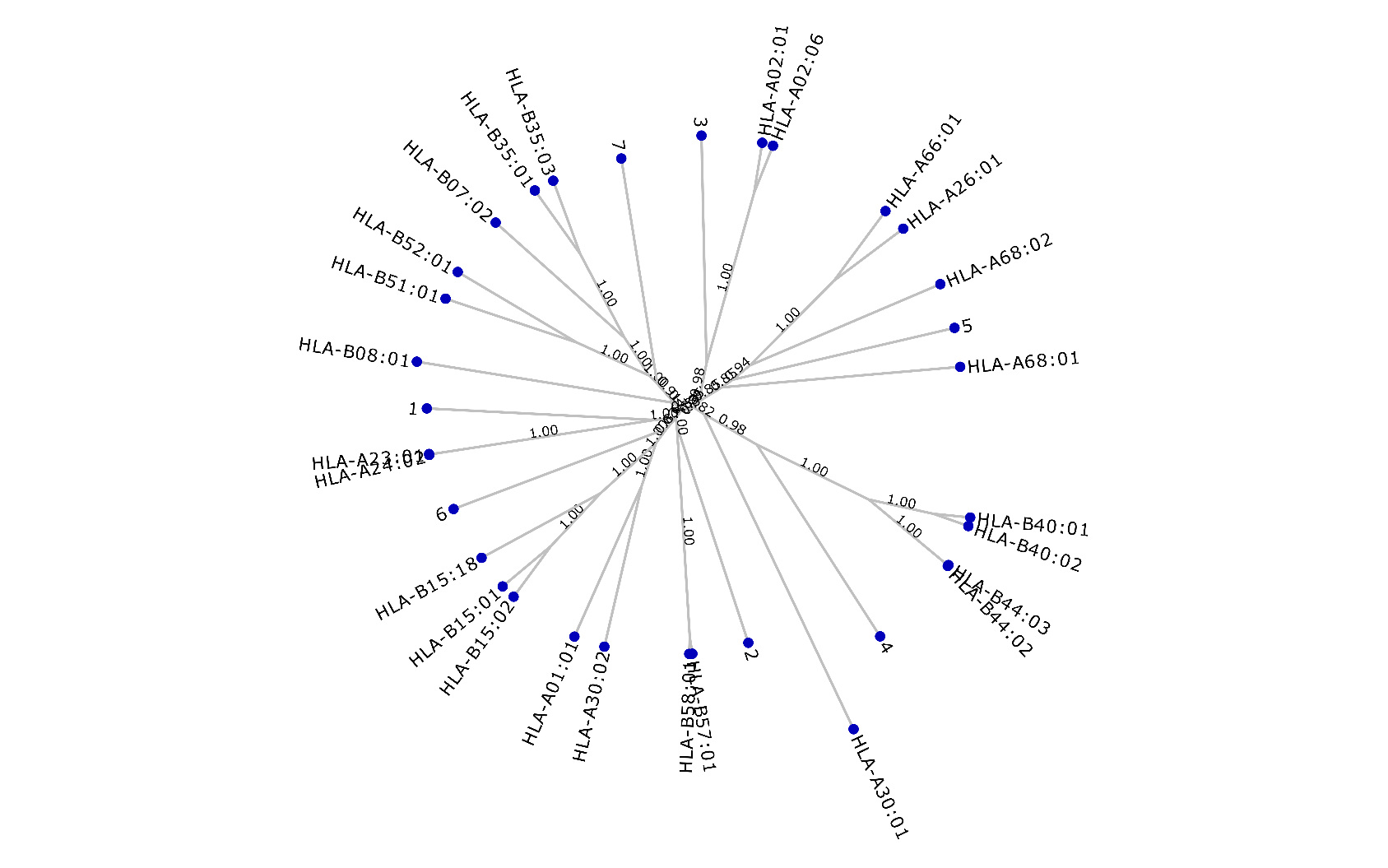
**Supplementary Figure S1**: Phylogenetic Tree generated for MHC-I epitopes and their respective HLAs

**Supplementary Figure S2**: Phylogenetic Tree generated for MHC-II epitopes and their respective HLAs


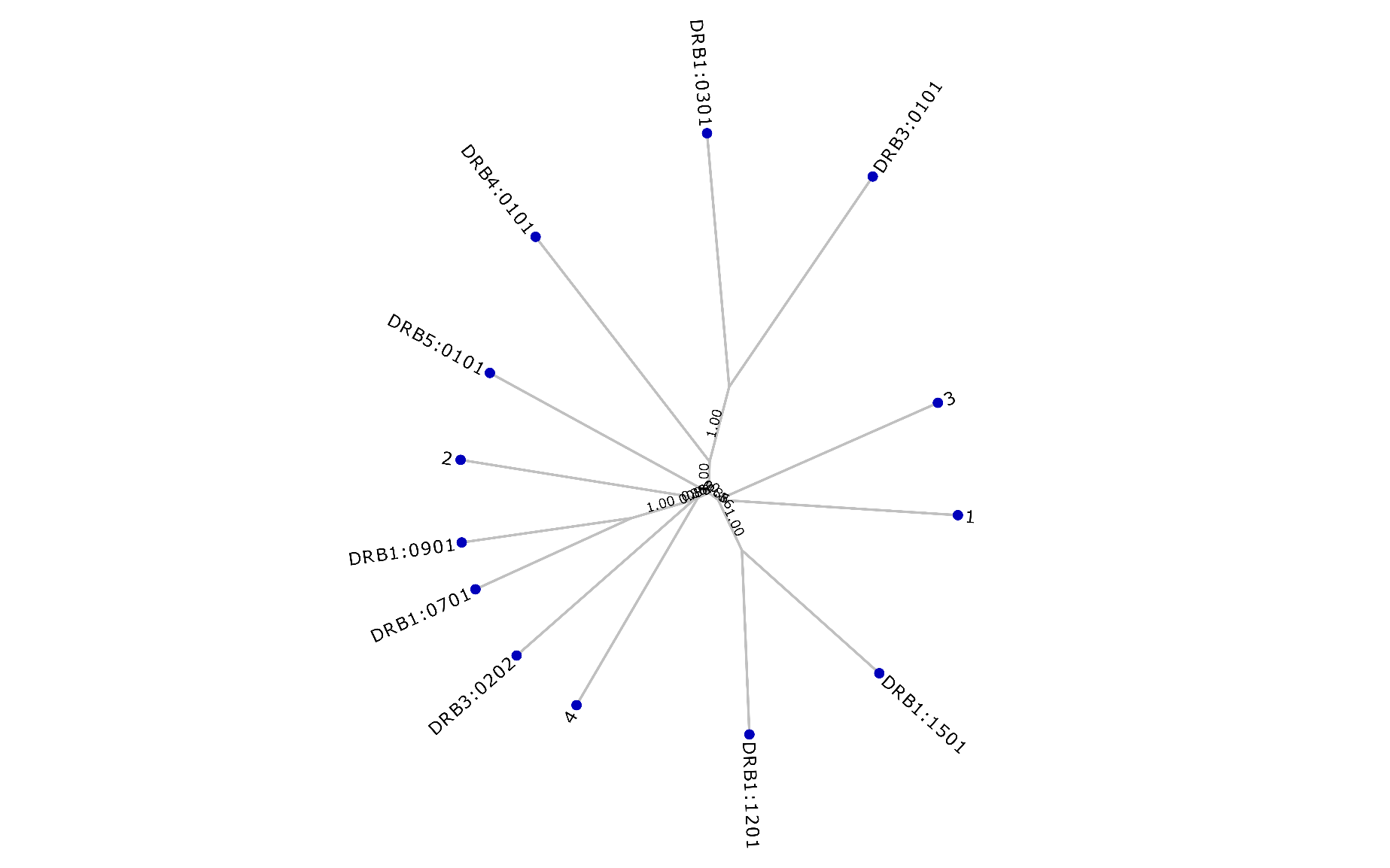
**Supplementary Figure S3:** (A) Modelled structure validation through Ramachandran Plot using PROCHECK showing 91% residues in favoured region, whereas, (B) Shows structure confirmation for final vaccine construct generated through PSIPRED nearly same position of
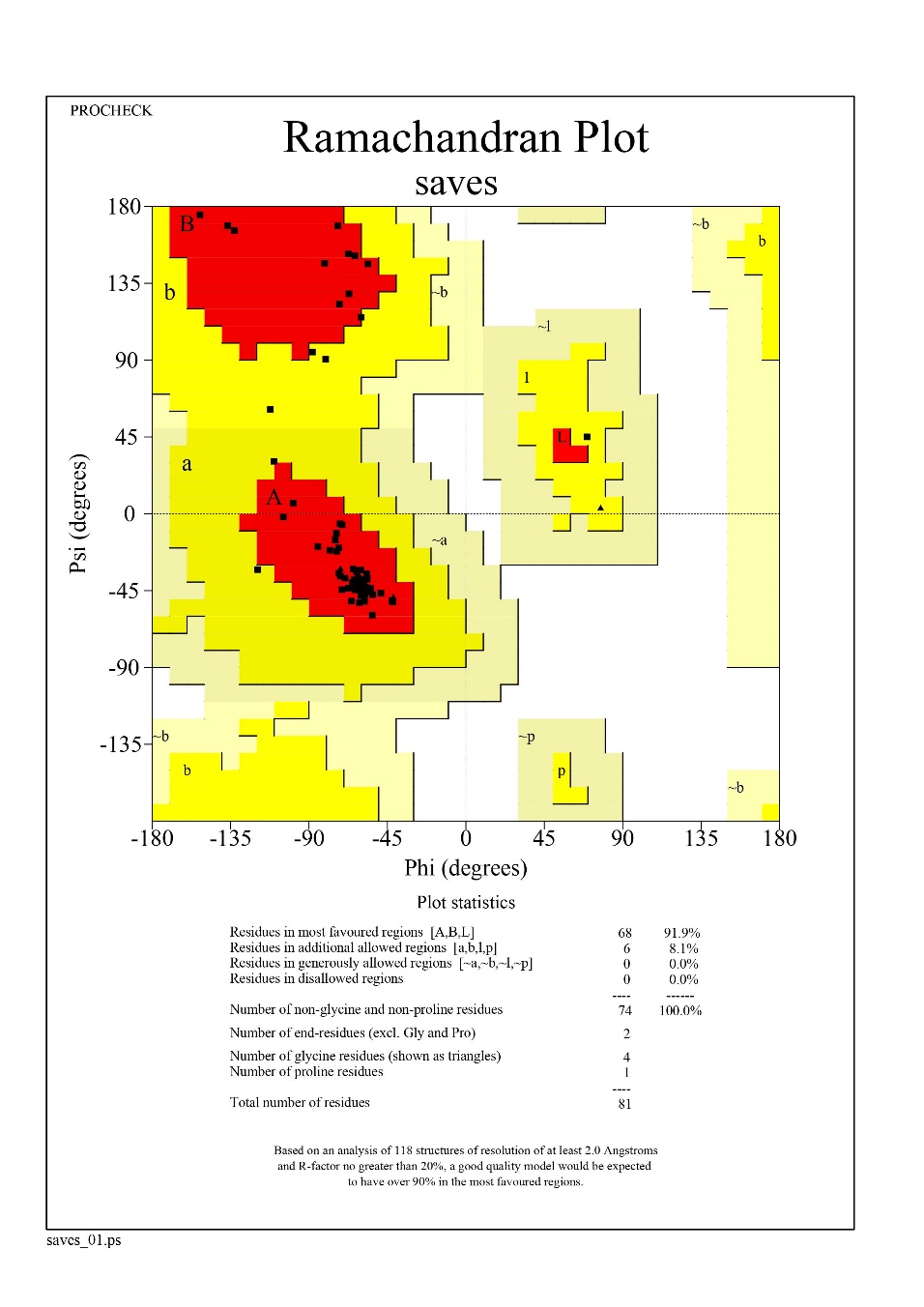
helixes and beta sheets as modelled structure.

B

A


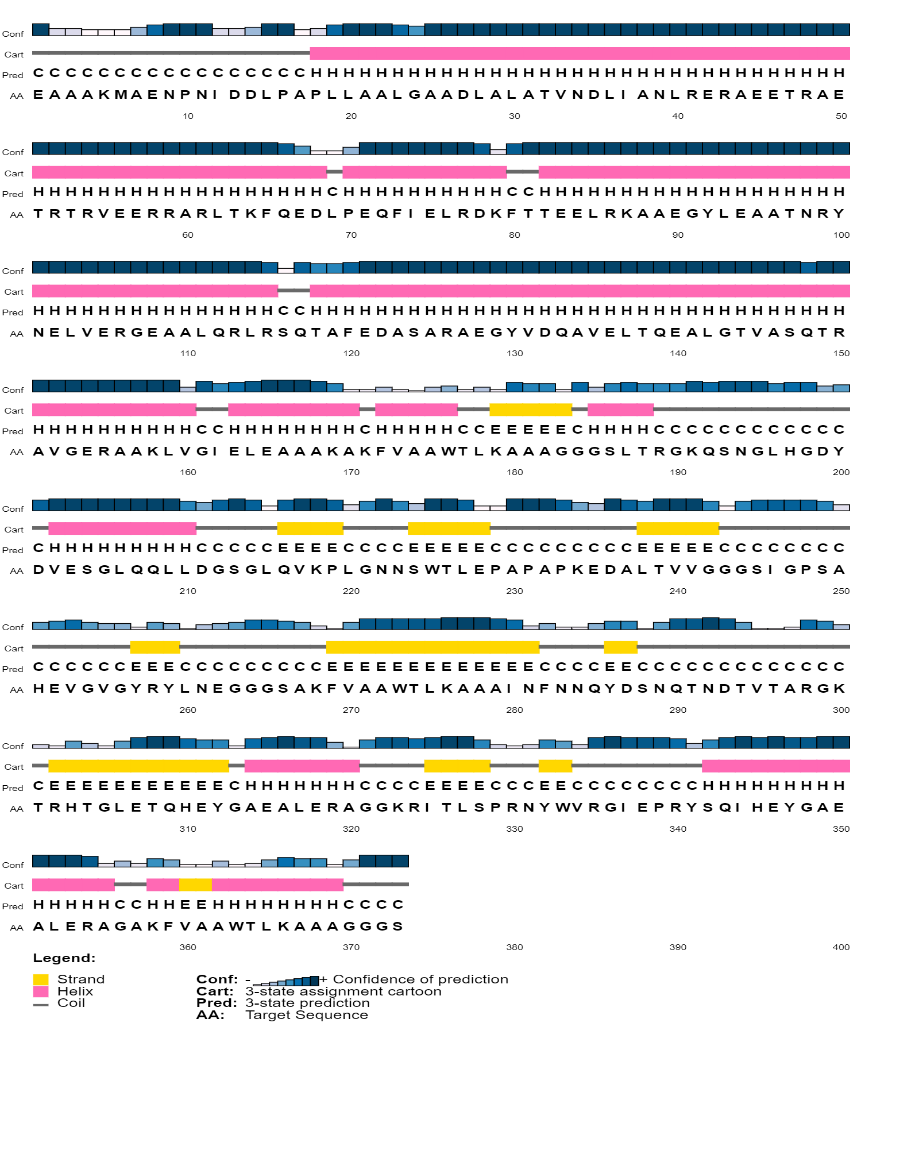


**
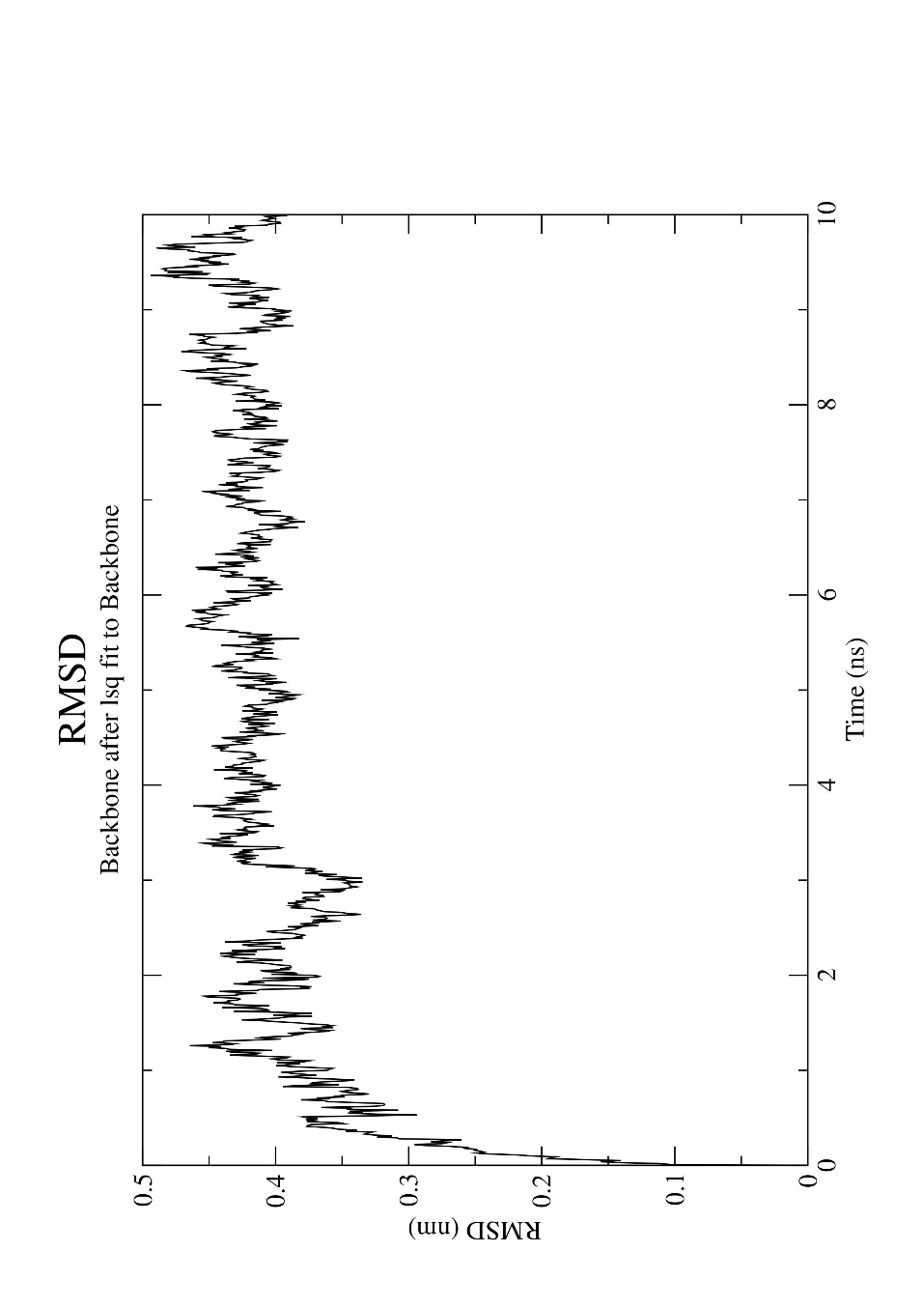
Supplementary Figure S4**. Molecular dynamics simulation of V9. (A) Root mean square deviation (RMSD) of protein backbone.

**Supplementary Figure S5.** The results of molecular dynamics simulation of vaccine construct and TLR4/MD docked complex. (A) Deformability B-factor, (B) eigenvalues, (C) variance (red color indicates individual variances and green color indicates cumulative variances), and (D) co-variance map.


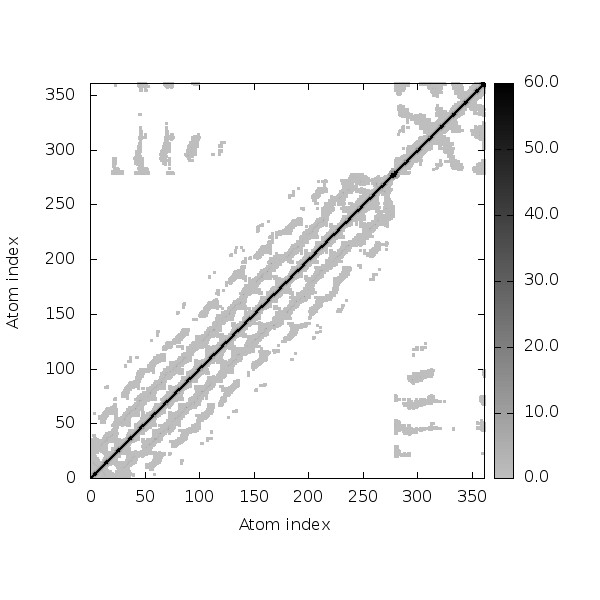

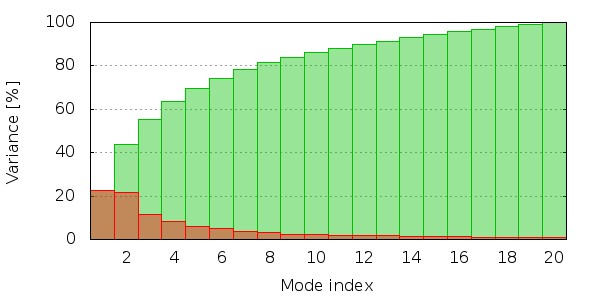

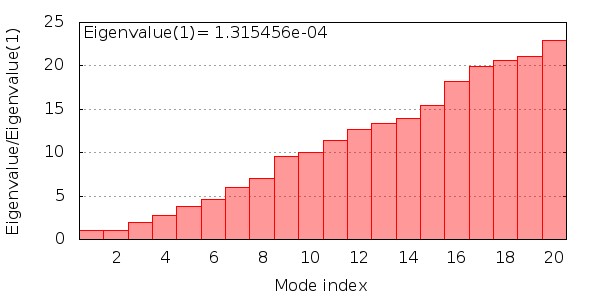

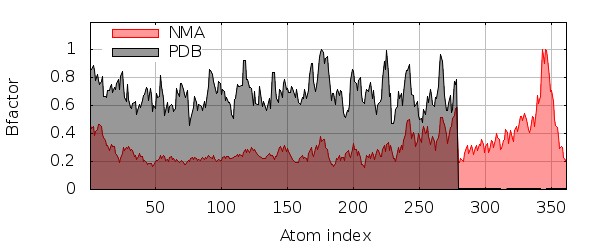


**A**

**B**

**D**

**C**


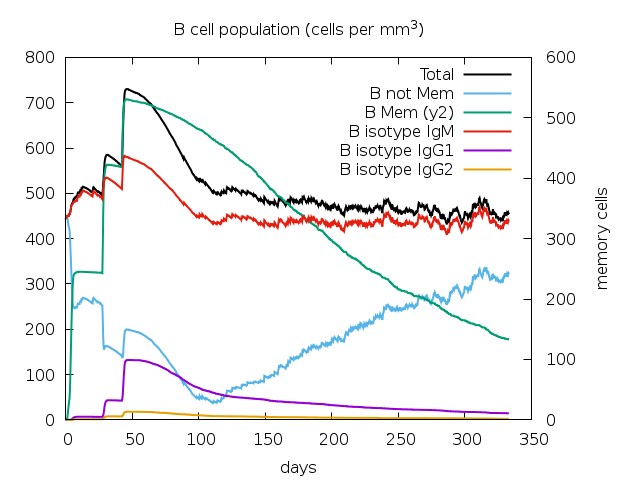
**Supplementary Figure S6.** C-ImmSim presentation of an in silico immune simulation with the construct. (A) Immunoglobulin production in response to antigen injections (black vertical lines); specific subclasses are showed as colored peaks and the evolution of B-cell populations after the three injections. (B) prediction of B cell population (C) T-helper cell populations per state after the injections. The resting state represents cells not presented with the antigen while the anergic state characterizes tolerance of the T-cells to the antigen due to repeated exposures. (D) Natural Killer cells production levels (E) Total production of T-cytotoxic cells, and (F) The main plot shows cytokine levels after the injections. The insert plot shows IL-2 level with the Simpson index, D shown by the dotted line. D is a measure of diversity. Increase in D over time indicates emergence of different epitope-specific dominant clones of T-cells. The smaller the D value, the lower the diversity


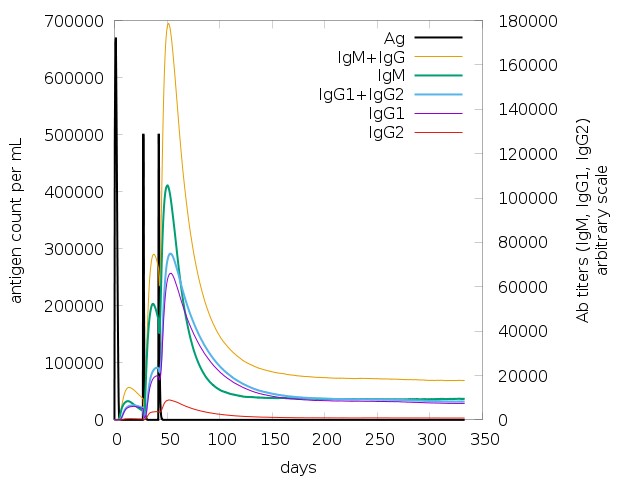


A


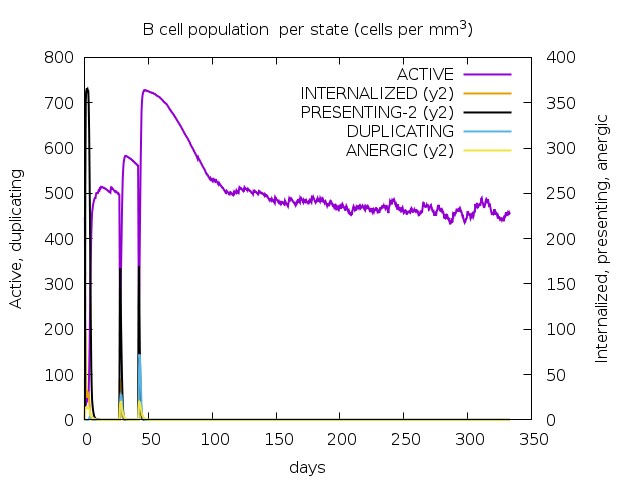


B

C


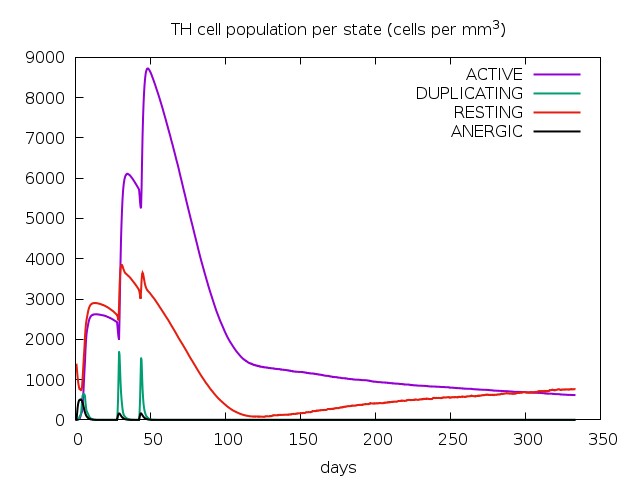

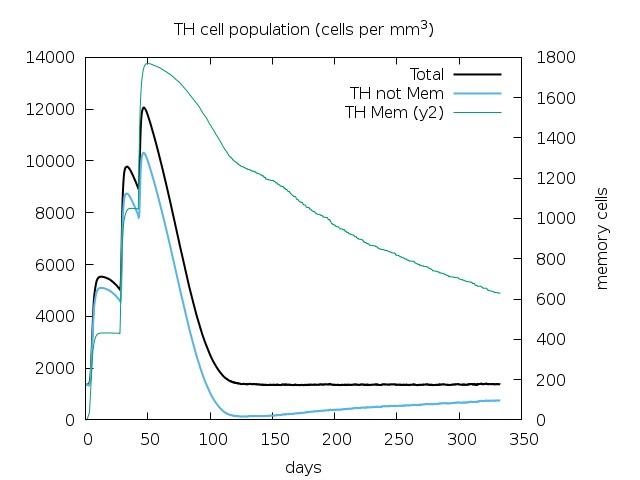


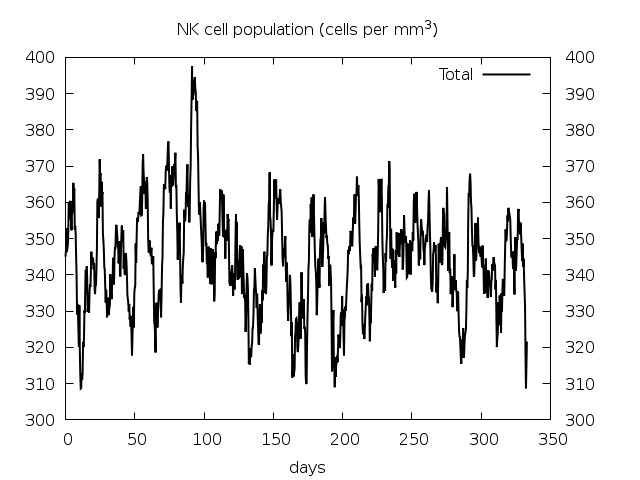


D

E


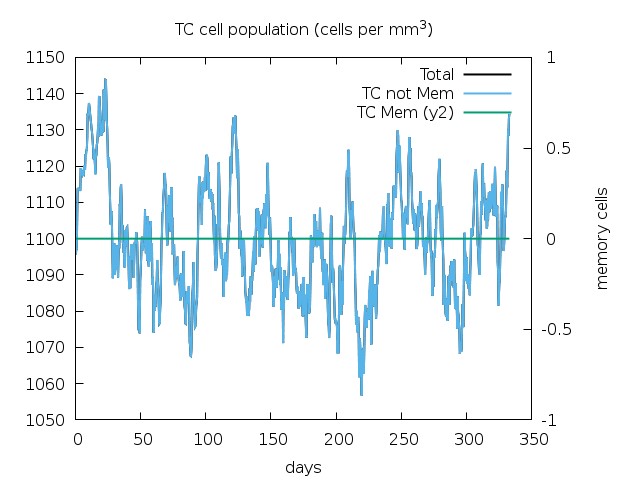

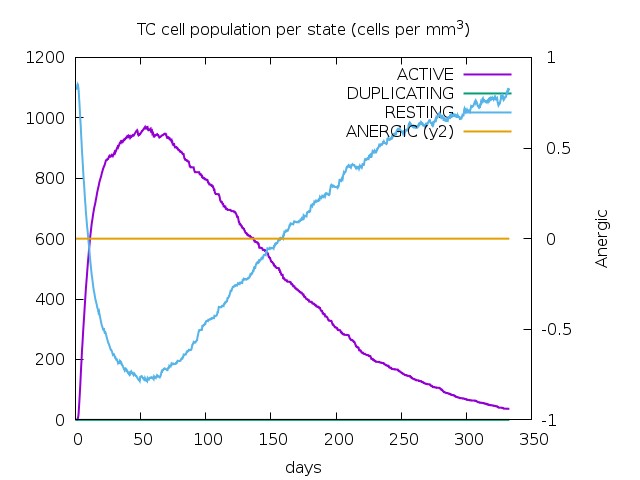


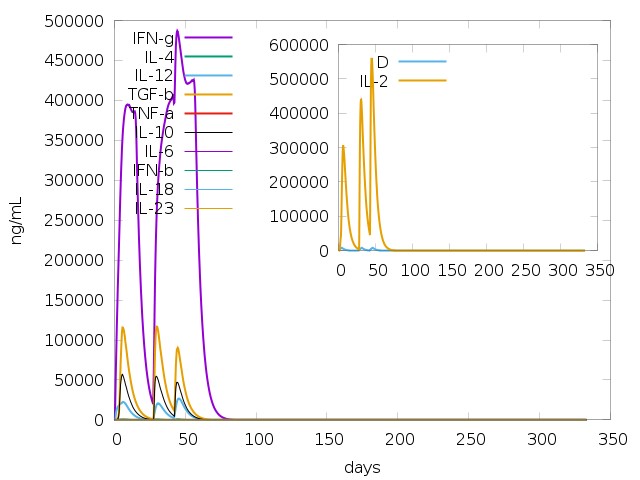


F
